# Comparative transcriptome among *Euscaphis konishii* Hayata tissues and analysis of genes involved in flavonoid biosynthesis and accumulation

**DOI:** 10.1186/s12864-018-5354-x

**Published:** 2019-01-09

**Authors:** Wenxian Liang, Lin Ni, Rebeca Carballar-Lejarazú, Xiaoxing Zou, Weihong Sun, Lingjiao Wu, Xueyuan Yuan, Yanling Mao, Wei Huang, Shuangquan Zou

**Affiliations:** 1 College of Forestry, Fujian Agriculture and Forestry University, Fuzhou, China; 2 Fujian Colleges and Universities Engineering Research Institute of Conservation & Utilization of Natural Bioresources, Fujian Agriculture and Forestry University, Fuzhou, China; 3 College of Plant Protection, Fujian Agriculture and Forestry University, Fuzhou, China; 4 Department of Microbiology & Molecular Genetics, University of California, Irvine, USA; 5 College of Resources and Environment, Fujian Agriculture and Forestry University, Fuzhou, China; 6 College of Life Sciences, Fujian Agriculture and Forestry University, Fuzhou, China

**Keywords:** *Euscaphis konishii* Hayata, Transcriptome, Gene expression, Flavonoid, Biosynthesis, Transport, Transcrip factor

## Abstract

**Bachground:**

*Euscaphis konishii* Hayata, a member of the Staphyleaceae Family, is a plant that has been widely used in Traditional Chinese Medicine and it has been the source for several types of flavonoids. To identify candidate genes involved in flavonoid biosynthesis and accumulation, we analyzed transcriptome data from three *E. konishii* tissues (leaf, branch and capsule) using Illumina Hiseq 2000 platform.

**Results:**

A total of 91.7, 100.3 and 100.1million clean reads were acquired for the leaf, branch and capsule, respectively; and 85,342 unigenes with a mean length of 893.60 bp and N50 length of 1307 nt were assembled using Trinity program. BLASTx analysis allowed to annotate 40,218 unigenes using public protein databases, including NR, KOG/COG/eggNOG, Swiss-Prot, KEGG and GO. A total of 14,291 (16.75%) unigenes were assigned to 128 KEGG pathways, and 900 unigenes were annotated into 22 KEGG secondary metabolites, including flavonoid biosynthesis. The structure enzymes involved in flavonoid biosynthesis, such as phenylalanine ammonia lyase, cinnamate 4-hydroxylase, 4-coumarate CoA ligase, shikimate O-hydroxycinnamoyltransferase, coumaroylquinate 3′-monooxygenase, caffeoyl-CoA O-methyltransferase, chalcone synthase, chalcone isomerase, flavanone 3-hydroxylase, flavonoid 3′-hydroxylase, flavonoid 3′,5′-hydroxylase, flavonolsynthese, dihydroflavonol 4-reductase, anthocyanidinreductase, leucoanthocyanidin dioxygenase, leucoanthocyanidin reductase, were identified in the transcriptome data, 40 UDP-glycosyltransferase (UGT), 122 Cytochrome P450 (CYP) and 25 O-methyltransferase (OMT) unigenes were also found. A total of 295 unigenes involved in flavonoid transport and 220 transcription factors (97 MYB, 84 bHLH and 39 WD40) were identified. Furthermore, their expression patterns among different tissues were analyzed by DESeq, the differentially expressed genes may play important roles in tissues-specific synthesis, accumulation and modification of flavonoids.

**Conclusion:**

We present here the de novo transcriptome analysis of *E. konishii* and the identification of candidate genes involved in biosynthesis and accumulation of flavonoid. In general, these results are an important resource for further research on gene expression, genomic and functional genomics in *E. konishii* and other related species.

**Electronic supplementary material:**

The online version of this article (10.1186/s12864-018-5354-x) contains supplementary material, which is available to authorized users.

## Background

The major active ingredients of medicinal plants are secondary metabolites, which their biosynthesis and accumulation are different among development stages, organs [1], environment [2], artificial tending measures and even different lineages [3]. The information provided from transcriptome studies of different experimental conditions or tissues can help in the characterization of important traits related to secondary metabolite formation and to test the molecular mechanisms associated to these metabolites [4–6]. *Euscaphis* is a valuable ornamental and medicinal plant from the Staphyleaceae Family, which contains two species in China—*Euscaphis japonica* Dippel and *Euscaphis konishiii* Hayata. It has been widely used to treat headaches, dizziness, colds, urticaria, hernia, and rheumatism according to Flora of Fujian Province. Until now, several kinds of compounds have been isolated from *Euscaphis*, such as triterpene compounds [7–10], phenolic acid compounds [11, 12], flavonoid compounds [9, 11] and others [11, 13, 14]. Anti-cancer, anti-inflammatory and antifibrotic activities have been demonstrated from *Euscaphis* extracts from different organs by modern pharmacological research, showing that the major active ingredients were triterpenes, flavonoids and phenolic acid [7–9, 11]. Several kinds of flavonoid compounds have been isolated from *E. konishii* capsule in our previous study (in press). However, the molecular mechanism of active ingredients biosynthesis and accumulation was still unclear because of the lack of *E. konishiii* genomic data.

Flavonoids are the most common and widely distributed polyphenolic secondary metabolites in plants. Flavonoids have many biochemical properties, such as antioxidant [15, 16], anticancer [17, 18], hepatoprotective [19, 20], antiviral [21, 22], anti-inflammatory [23, 24] and antibacterial activity [17, 25]. In response to the abundant biochemical activities, research groups have directed their attention to the flavonoid biosynthesis and accumulation mechanism in plants. Until now, the biosynthesis pathway of flavonoids has been well studied in some plants such as *Vaccinium macrocarpon* Ait. [26] and *Dracaena cambodiana* [27]. Given the variable distribution and contents of those flavonoids among different plants, organs, origins and even lineages, the molecular mechanisms of flavonoid biosynthesis, transport and regulation might be diverse and complex. Consequently, it is necessary to study the molecular mechanism of flavonoids biosynthesis and regulation in *E. konishii*. However, the lack of genomic and transcriptomic data makes difficult to study these mechanisms in *E. konishii*.

RNA sequencing (RNA-seq), which uses next-generation sequencing (NGS) to reveal the presence and quantity of RNA in a biological sample at a given moment in time [28], have been widely used as a powerful and valid tool to reveal gene expression patterns of any given condition due to high-throughput, accuracy and reproducibility [29]. In this study, high-throughput sequencing was performed to describe the transcriptome of *E. konishiii* in three different tissues to further understand the molecular mechanism of flavonoid biosynthesis.

## Results

### Rutin quantitative determination in *E. konishii* tissues

**Fig. 1:**
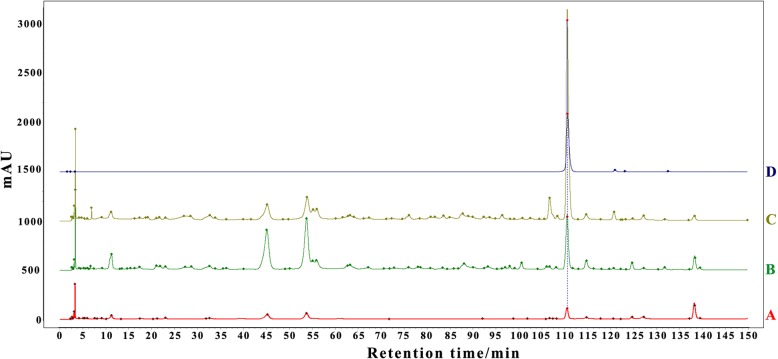
Typical chromatograms for determining rutin in three organs of *E. konishii*. (A) Branch; (B) Capsule; (C) Leaf; (D) Rtuin

Rutin content in different *E. konishii* tissues was determined by HPLC, results are shown in Table 1 and  Fig. 1. Quercetin-3-*O*-rutinoside (rutin) quantification shows that there is significant difference among different tissues. Leaf showed the highest content of rutin with 26.31 ± 2.43 mg·g^− 1^, while the branch showed the lowest rutin amount (1.43 ± 0.16 mg·g^− 1^).

**Table 1:** Quercetin-3-*O*-rutinoside (rutin) contents in three *E. konishii* tissues

	Leaf	Branch	Capsule
Rutin content/mg·g^− 1^	26.31 ± 2.43 a	1.43 ± 0.16 c	7.29 ± 0.89 b

Data are represented as mean ± SD, different words indicate significant difference of the rutin content based on three biological replications. (*P < 0.05*, t-test; n  =  3)

### Transcript sequencing and assembly

**Fig. 2:**
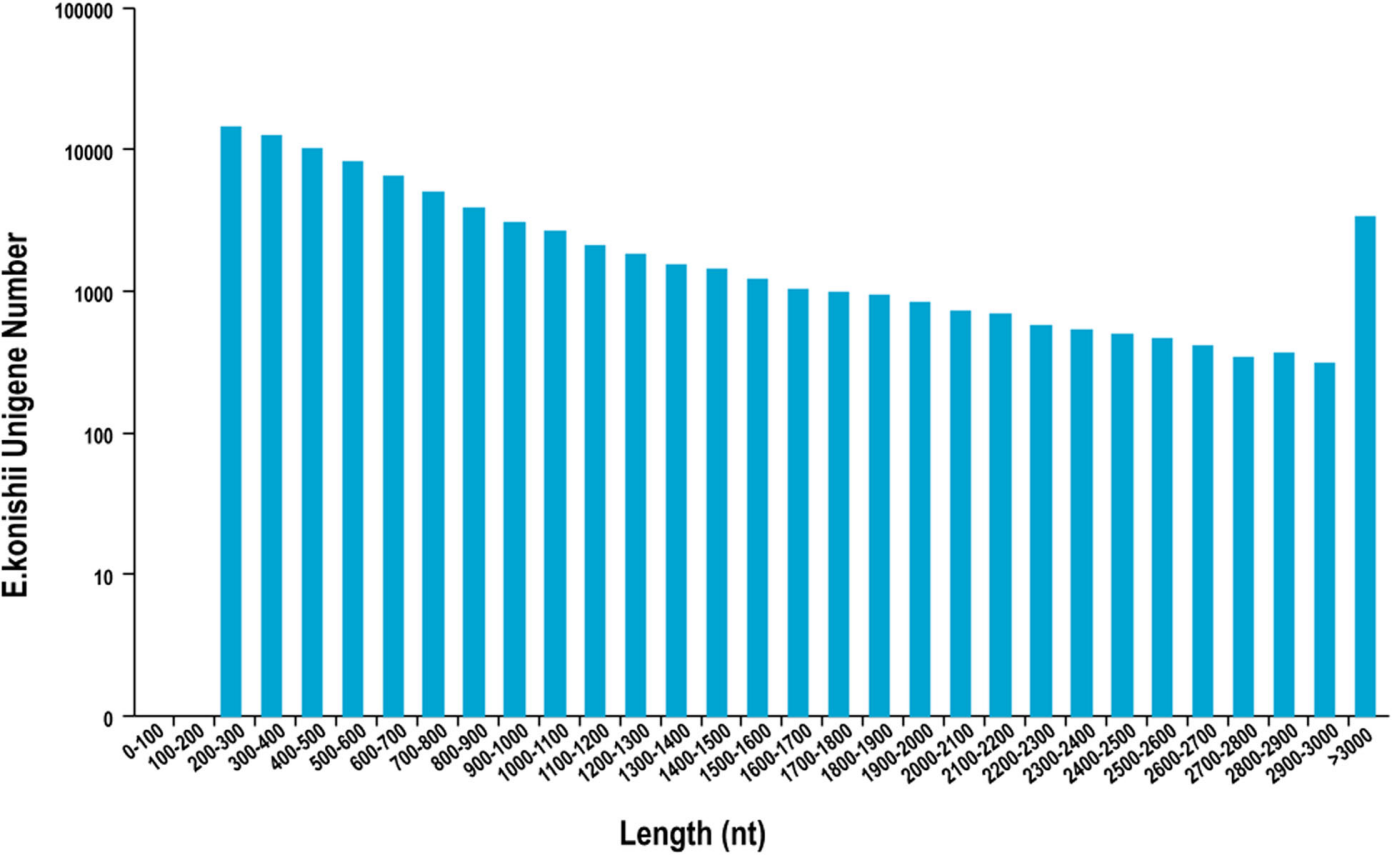
Unigenes length distribution.The y-axis number has been converted into logarithmic scale

Three mRNA libraries were generated from leaf, branch and capsule of *E. konishii* using Illumia sequencing technology. Adaptor sequences and low-quality reads were removed, approximately 91.7, 100.3 and 100.1 million clean reads were acquired; moreover, 27.11, 29.61 and 29.63 gigabase pairs of nucleotides were generated and the GC percentages were 45.13, 45.62 and 45.10 for leaf, branch and capsule, respectively (Table 2). Finally, a total of 85,342 unigenes with a mean length of 893.60 bp and N50 length of 1307 nt were assembled by Trinity program from the three tissues. The length distribution of all unigenes are shown in Fig. 2. The raw data has been submitted to NCBI, Sequence Read Archive (SRA) submission: SUB4637004.

**Table 2:** Summary of the assembly and annotation of the transcriptome

	Leaf	Branch	Capsule
Clean reads	91,768,881	100,363,012	100,124,694
Clean nucleotides(nt)	27,117,135,514	29,612,649,608	29,636,461,864
GC percentage (%)	45.13	45.62	45.10
Combined non-redundant unigene		85,342	
Total length		76,261,599	
Mean length		893.60	
N50(nt)		1307	
Nr		39,074 (45.79%)	
GO		23,899 (28%)	
COG		11,713 (13.72%)	
Swiss-Prot		24,234 (28.40%)	
KEGG		14,291 (16.75%)	
All annotated		40,218 (47.13%)

### Function annotation

The assembled unigenes were searched against NCBI non-redundant (Nr), Swiss-Prot, KEGG (Kyoto Encyclopedia of Genes and Genomes) pathway, GO (Gene Ontology) and COG (Clusters of Orthologous Groups of proteins) databases using BLAST (e < 10^− 5^). A total of 40,218 (47.13% of 85,342) unigenes were annotated to one or more functions from these databases, 39,074 (45.79%), 23,899 (28%), 11,713 (13.72%), 24,234 (28.40%), 14,291(16.75%) from Nr database, GO, COG, Swiss-Prot and KEGG databases, respectively (Table 2).

**Fig. 3:**
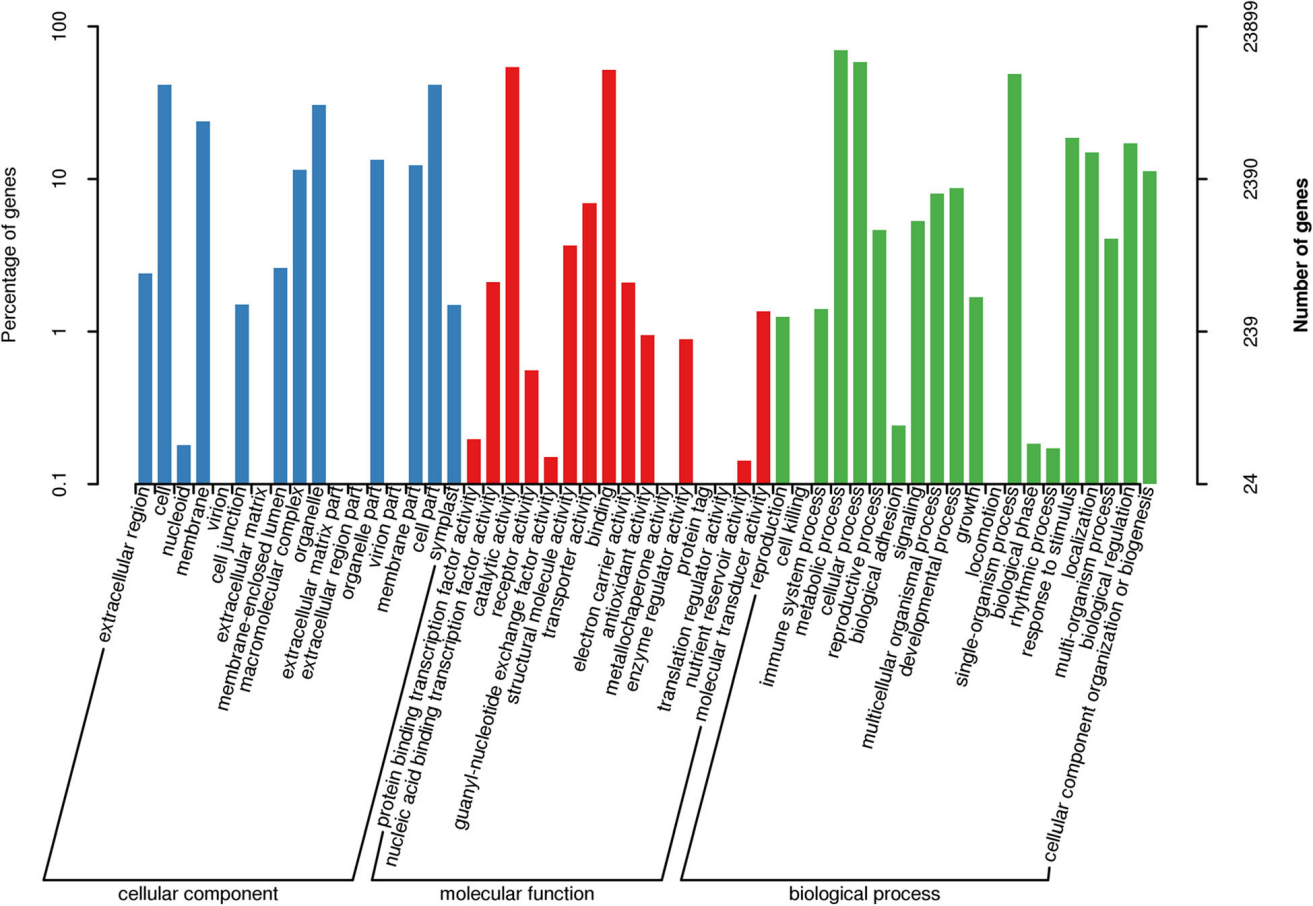
Gene ontology classification of *E. konishiii* transcriptome. Unigenes were annotated in three categories: cellular components, molecular functions, and biological process. Right y-axis indicates the number of genes in a category; left y-axis indicates the genes percentage in a specific

A total of 23,899 (28%) unigenes were annotated into GO terms (Fig. 3). The unigenes were classified into 54 subcategories within three standard categories (molecular functions, biological processes and cellular components). “Cell” and “cell part” were the top terms in the cellular process domain, in molecular function category, “catalytic activity” and “bingding” were the highest enriched, while “metabolic process” and “cellular process” were the most enriched in the biological process domain.

**Fig. 4:**
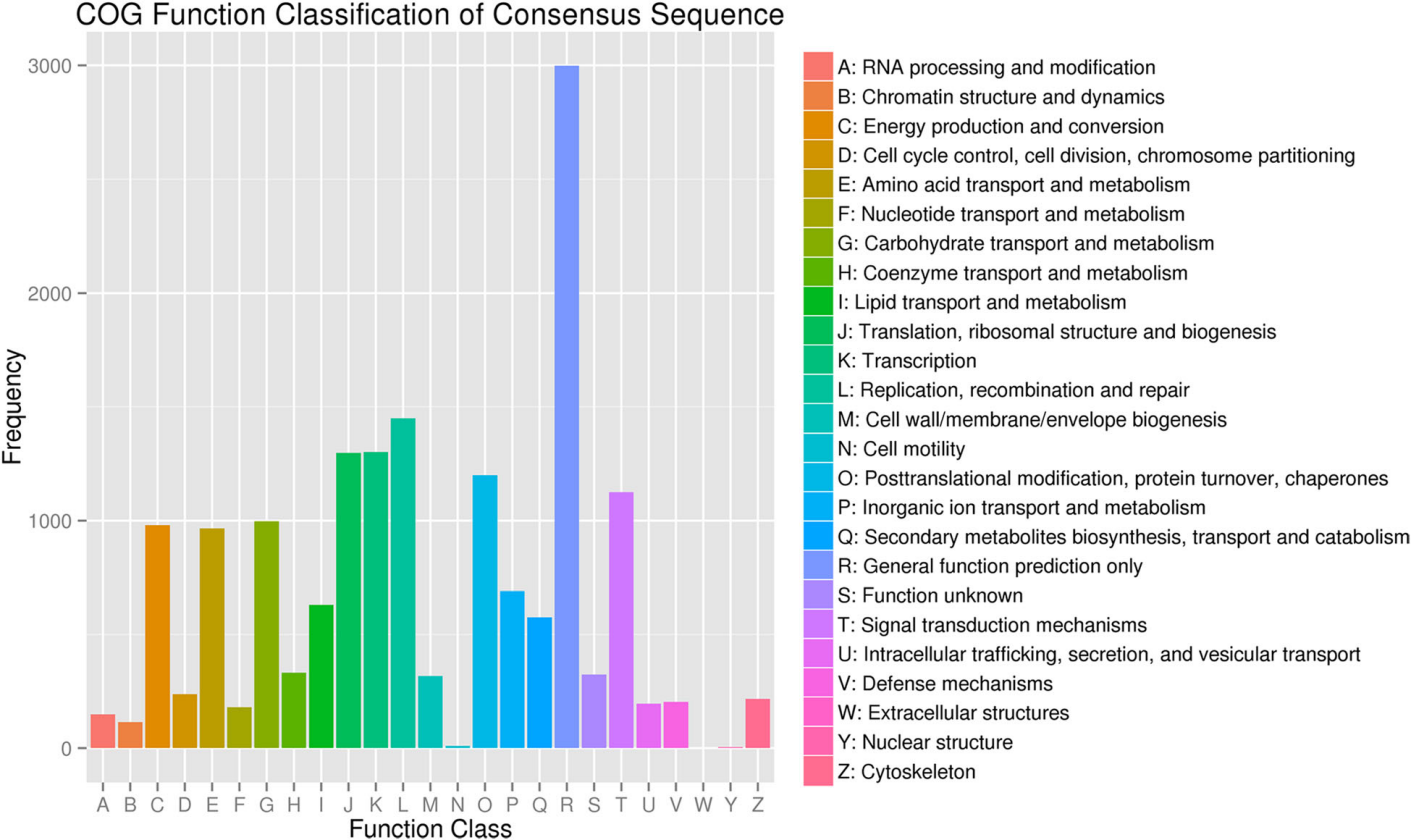
COG function classification of *E. konishiii* unigenes. All putative unigenes were analyzed using the COG database. COG classifications were divided into 26 functional categories; 11,713 classified unigenes were assigned to 25 COG classifications

COGs analysis results showed a total of 11,713 (13.72%) unigenes annotated to COG functional classes. “General function prediction only” was the largest group, followed by “replication, recombination and repair”, “transcription”, “translation, ribosomal structure and biogenesis”, “posttranslational modification, protein turnover, chaperones”, “signal transduction mechanisms”, “carbohydrate transport and metabolism” and “energy production and conversion” (Fig. 4).

KEGG (Kyoto Encyclopedia of Genes and Genomes) pathway analysis in this study was performed to identify biochemical pathway active in the branch, capsule and leaf of *E. konishii*. A total of 14,291 (16.75%) unigenes were assigned to 128 KEGG pathways (Additional file 1), covering five major KEGG categories. In the biosynthesis of secondary metabolites category, 900 unigenes were annotated into 22 KEGG secondary metabolites, “phenylpropanoid biosynthesis pathway” was the largest group, followed by “terpenoid backbone biosynthesis”, “steroid biosynthesis”, “carotenoid biosynthesis”, “tropane, piperidine and pyridine alkaloid biosynthesis”, “arachidonic acid metabolism”, “linoleic acid metabolism” and “flavonoid biosynthesis”.

### Differential gene expression among tissues

**Fig. 5:**
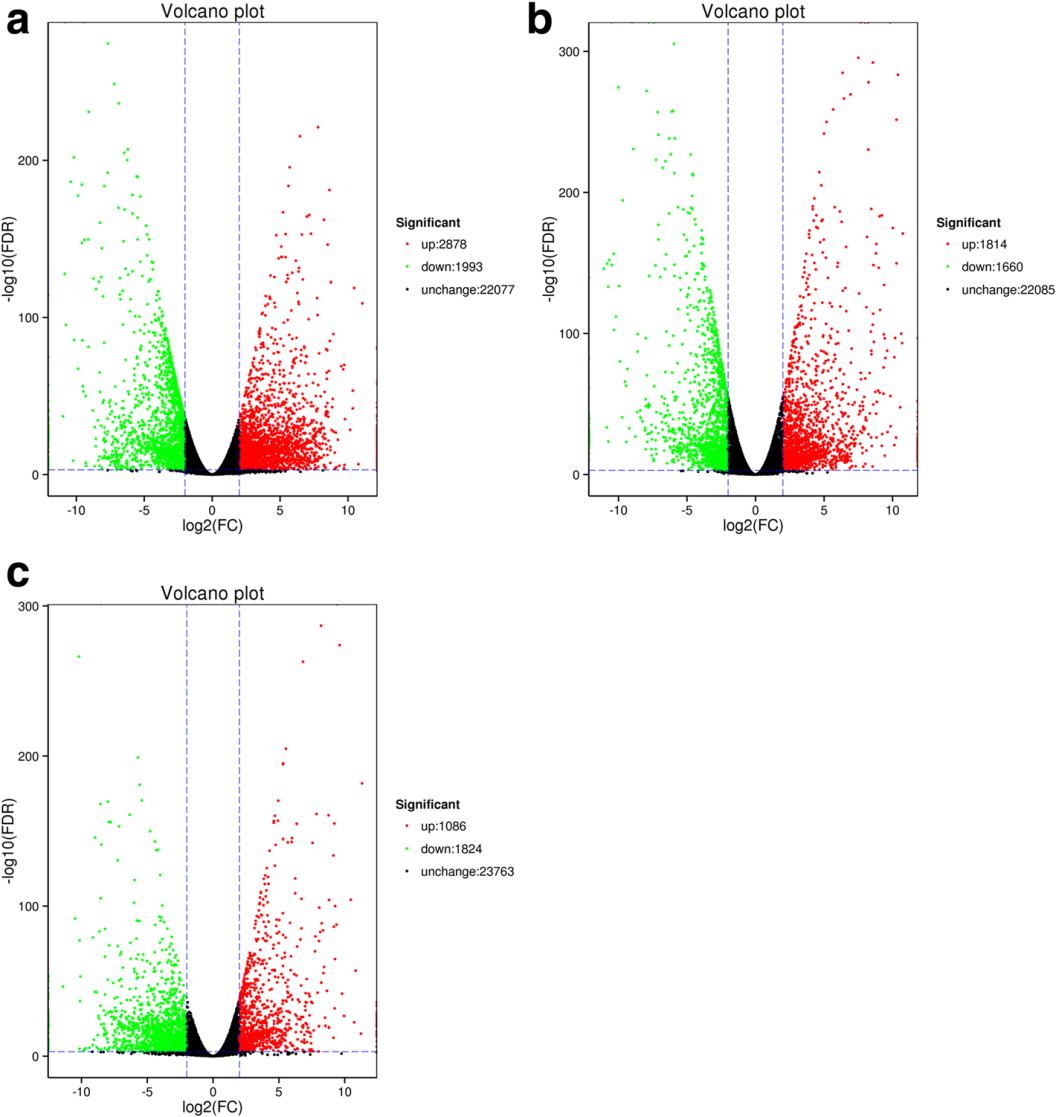
Gene differential expression in *E. konishiii* transcriptome. Volcano plot shows the differential expressed genes. (**a**) Leaf *vs* Branch, (**b**) Leaf *vs* Capsule, (**c**) Branch *vs* Capsule. (black spot: unchanged unigenes, green spot: down-regulated unigenes and red spot: represent up-regulated unigenes)

Differential expressed genes between two groups were identified by DESeq. A total of 4871 genes were differentially expressed between leaf and branch (2878 up-regulated and 1993 down-regulated in branch compared with leaf), the number of DEG between leaf *vs* capsule and branch *vs* capsule was 3474 (1814 up-regulated and 1660 down-regulated) and 2910 (1086 up-regulated and 1824 down-regulated), respectively (Table 3, Fig. 5).

**Table 3:** Statistical table of differently expressed genes (DEGs) and annotation

Type	Leaf *vs* Branch	Leaf *vs* Capsule	Branch *vs* Capsule
number	4871	3474	2910
up-regulated	2878	1814	1086
down-regulated	1993	1660	1824
COG	1178	825	706
GO	2525	1786	1571
KEGG	1247	877	777
Swiss-port	2833	1996	1816
Nr	3889	2738	2414
All annotated	3928	2755	2429

### GO enrichment and KEGG pathway analysis of DEGs

To further identify the biological functions of the DEGs, the DEGs were blast against GO and KEGG databases. A total of 2515, 1786 and 1571 DEGs were annotated from leaf *vs* branch, leaf *vs* capsule, and branch *vs* capsule, respectively. “Catalytic activity”, “cellular process”, “single-organism process”, “organic substance metabolic process, binding and primary metabolic process” were the most enriched GO terms in leaf-branch DEGs; while in leaf-capsule DEGs, “metabolic process”, “cellular process”, “single-organism process”, “organic substance metabolic process”, “primary metabolic process” and “cell part” were the most annotated terms; and in branch-capsule the “metabolic process”, “catalytic activity”, “single-organism process”, “cellular process”, “binding and organic substance metabolic process” were the most abundant terms (Additional file 2).

In leaf-branch DEGs, 1247 unigenes were mapped to 109 KEGG pathways, and the most significantly enriched metabolic were “phenylpropanoid biosynthesis”, “cyanoamino acid metabolism”, “carotenoid biosynthesis”, “starch and sucrose metabolism”, “plant hormone signal transduction, photosynthesis” and “flavonoid biosynthesis”. In contrast, leaf-capsule DEGs, comprised 877 unigenes that were assigned into 108 KEGG pathways: “photosynthesis, phenylalanine metabolism”, “phenylpropanoid biosynthesis”, “cyanoamino acid metabolism”, “flavonoid biosynthesis”, “photosynthesis- antenna proteins”, “carotenoid biosynthesis”, “pentose and glucuronate interconversions”, “diterpenoid biosynthesis”, “glycine, serine and threonine metabolism” were the most significantly enriched pathways. Finally, branch-capsule DEGs, comprised 777 unigenes that were represented in 107 KEGG pathways, and “plant hormone signal transduction”, “diterpenoid biosynthesis”, “phenylalanine metabolism”, “starch and sucrose metabolism”, “taurine and hypotaurine metabolism”, “carotenoid biosynthesis”, “cyanoamino acid metabolism”, “isoflavonoid biosynthesis”, “phenylpropanoid biosynthesis”, “galactose metabolism” and “pentose and glucuronate interconversions” were the most significantly enriched pathways (Additional file 3).

### Candidate genes involved in flavonoid biosynthesis

Given that *E. konishiii* is rich in flavonoids, candidate genes involved in flavonoids biosynthesis were identified in this study. In plants, the flavonoids biosynthesis pathway has been studied in several species, such as the model plant *Arabidopsis thaliana* [30], important crops like *Vitis vinifera* [31, 32], *Zea mays* [33] and *Hordeum vulgare* [34]. However, the mechanism of flavonoids biosynthesis in *E. konishiii* is still not understood.

**Fig. 6:**
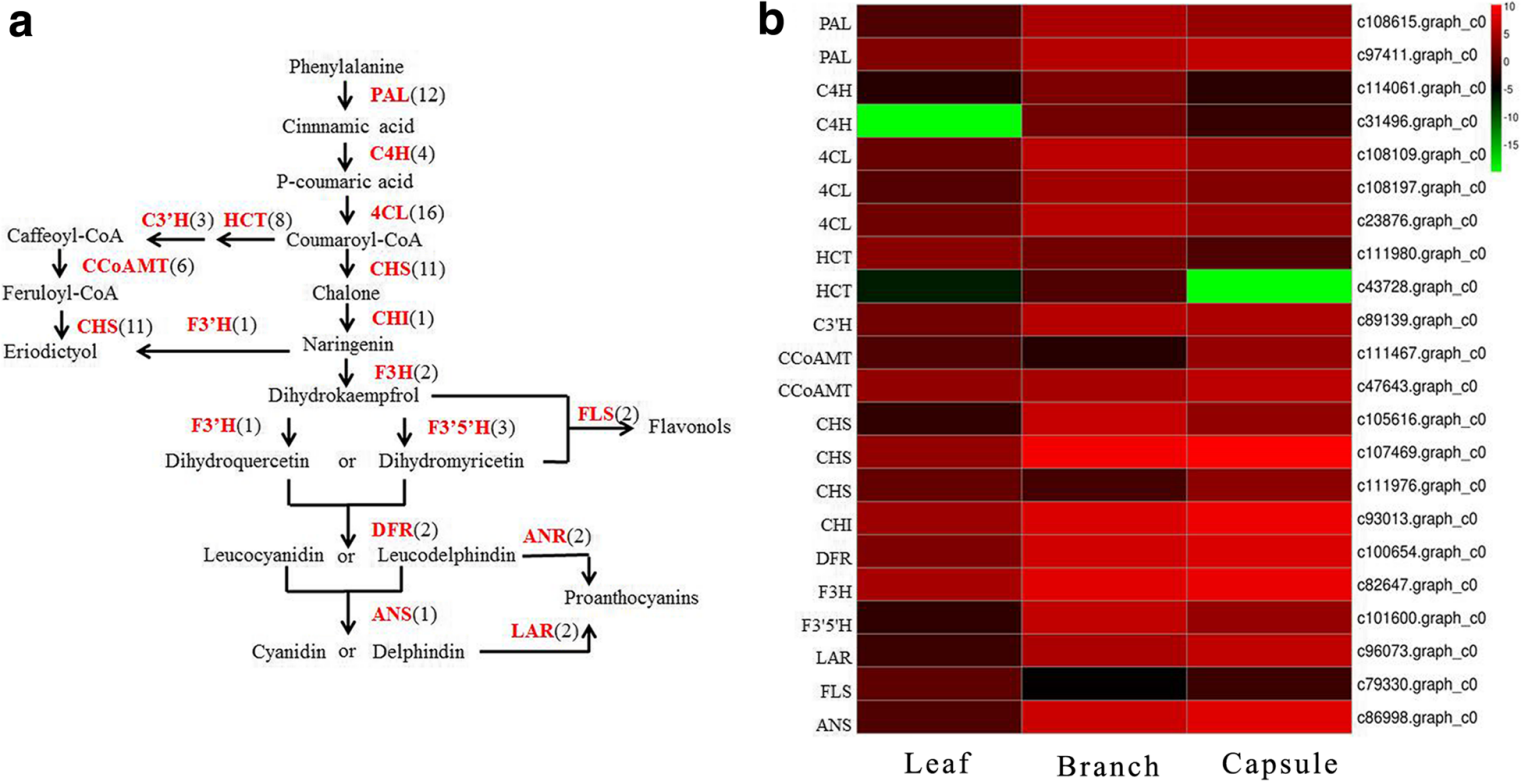
Putative flavonoids biosynthesis pathway in *E. konishiii*. (**a**) Pathway for flavonoids biosynthesis. The numbers in brackets following each gene name indicate the number of *E. konishiii* unigenes corresponding to that gene. Enzyme abbreviations are as follows: PAL, phenylalanine ammonia lyase; C4H, cinnamate 4-hydroxylase; 4CL, 4-coumarate CoA ligase; HCT, shikimate O-hydroxycinnamoyltransferase; C3’H, coumaroylquinate 3′-monooxygenase; CCoAMT, caffeoyl-CoA O-methyltransferase; CHS, chalcone synthase; CHI, chalcone isomerase; F3H, flavanone 3-hydroxylase; F3’H, flavonoid 3′-hydroxylase; F3’5’H, flavonoid 3′,5′-hydroxylase; FLS, flavonolsynthese; DFR, dihydroflavonol 4-reductase; ANR, anthocyanidinreductase; ANS, leucoanthocyanidin dioxygenase; LAR, leucoanthocyanidin reductase. (**b**) Expression levels of candidate *E. konishiii* unigenes coding for key enzymes involved in flavonoids biosynthesis pathways. Green and red colors are used to represent low-to-high expression levels, and color scales correspond to the mean centered log_2_-transformed FPKM values

We identified the genes encoding the enzymes involved in flavonoid biosynthesis pathway in the annotated *E. konishiii* transcriptome. A brief schematic of flavonoids biosynthesis pathway is shown in Fig. 6a, which was modified from KEGG databases. Flavonoids are synthesized in the cytosol from Coumaroyl-CoA, which is synthesized from phenylalanine by the enzymes phenylalanine ammonia-lyase (PAL, 12 unigenes), cinnamate 4-hydroxylase (C4H, 4 unigenes) and 4 coumarate CoA ligase (4CL,16 unigenes). Coumaroyl-CoA can be converted either to naringenin under the action of chalcone synthase (CHS, 11 unigenes) and chalcone isomerase (CHI, 1 unigene) or to eriodictyol by the enzymes shikimate O-hydroxycinnamoyltransferase (HCT, 8 unigenes), coumaroylquinate3′-monooxygenase (C3’H, 3 unigenes), caffeoyl-CoA O-methyltransferase (CCoAMT, 6 unigenes) and chalcone synthase (CHS, 11 unigenes); flavanone 3-hydroxylase (F3H, 2 unigenes) converts the naringenin to dihydrokaempferols (DHK) and then are catalyzed to dihydroquercetins (DHQ) by flavonoid 3’-hydroxylase (F3’H, 1 unigene) or dihydromyricetins (DHM) by flavonoid 3′,5′-hydroxylase (F3’5’H, 3 unigenes). Finally, DHK, DHM and DHQ are converted to flavonols by flavonolsynthese (FLS, 2 unigenes). In the anthocyanin branch, dihydroflavonol 4-reductase (DFR, 2 unigenes) catalyzes DHQ and DHM to leucocyanidins and leucodelphinidins and then continually converted to cyanidin or delphindin by leucoanthocyanidin dioxygenase (ANS, 1 unigene). The modification reactions, such as glycosylation, hydroxylation and methylation by enzymes UDP-glycosyltransferase (UGT) [35], Cytochrome P450 (CYP) and O-methyltransferase (OMT) generate a variety of flavonoids. In this study, 40 UGT, 122 CYP and 25 OMT unigenes were found.

Furthermore, the expression patterns of all the transcripts encoding enzymes involved in the flavonoids biosynthesis steps were analyzed. 2 PAL, 2 C4H, 3 4CL, 2 HCT, 1 C3’H, 2 CHS, 1 CHI, 1 F3H, 1 F3’5’H, 1 LAR, 1 ANS, 20 CYP and 5 OMT unigenes were up-regulated and 1 CHS, 1 FLS, 8 UGT, 25 CYP and 1 OMT unigenes were down regulated in branch against in leaf. In capsule against leaf, 2 PAL, 3 4CL, 1 HCT, 1 C3’H, 2 CHS, 1 CHI, 1 F3H, 1 F3’5’H, 1 LAR,1 ANS, 7 UGT, 16 CYP and 5 OMT unigenes were up-regulated and 1 HCT, 1 FLS, 2 UGT, 9 CYP and 2 OMT unigenes were down regulated. In capsule against branch, 1 CHS, 2 CCoAMT, 11 UGT, 21 CYP and 2 OMT unigenes were up-regulated and 2 C4H, 2 HCT, 1 CHS, 1 F3’5’H, 2 UGT, 13 CYP and 2 OMT unigenes were down-regulated (Fig. 6b, Additional file 4). Differential gene expression among different tissues might play important roles in flavonoid biosynthesis in *E. konishiii*.

### Candidate genes involved in flavonoid transport

In plants, flavonoids are synthesized in the cytosol and then stored into vacuoles for storage or to be transported to other locations [36]. According to Zhao et al. (2010), there are three major mechanisms proposed for flavonoid transport: membrane vesicle-mediated transport (MVT), membrane transporter-mediated transport (MMT) and glutathione-S-transferases (GST) [37]. Several genes responsible for these mechanisms have been identified in *Arabidopsis* [38, 39], *M. truncatula* [40], *Zea mays* [41] and *V. vinifera* [42, 43].

ATP-binding cassette transporters (ABC) (G-type (APCG) and the multidrug resistance-associated protein (MRP)-type), H^+^-ATPases, multidrug and toxic compound extrusion protein (MATE) transporters and H^+^-Ppase belong to MMT, while solube N-ethylmaleimidesensitive factor attachment protein receptors (SNARE) and vacuolar sorting receptor (VSR) are responsible for MVT. In *E. konishiii*, we found 29 unigenes encoding MATE, 175unigenes encoding MRP/ABCG, 19 unigenes encoding H^+^-ATPase, 56 unigenes encoding GST, 14 unigenes encoding SNARE and 2 unigenes encoding VSR. Expression patterns of all the above described genes were analyzed, moreover, 24 ABC/MRP, 2 GST, 1 H + -ATPase and 2 MATE unigenes were up-regulated and 7ABC/MRP, 4 GST, 1 H + -ATPase, 2 MATE unigenes were down-regulated in branch *vs* leaf. In capsule *vs* leaf,13 ABC/MRP, 6 GST, 1 H + -ATPase and 2 MATE unigenes were up-regulated and 7 ABC/MRP, 2 GST, and 2 MATE unigenes were down-regulated. Finally, 9 ABC/MRP, 6 GST and 2 MATE unigenes were up-regulated and 10 ABC/MRP, 2 MATE unigenes were down-regulated in capsule *vs* branch (Additional file 4).

### Candidate transcription factors involved in flavonoid biosynthesis and transport

Recently, it has been showed that structural genes involved in flavonoids are controlled by MYB protein family, basic helix-loop-helix (bHLH) transcription factors (TFs) and WD-repeat-containing proteins [44, 45]. MYB TFs are one of the largest TF families and plays important roles in controlling cellular processes, such as development, responses to biotic and abiotic stresses [46], differentiation [47] and metabolism [48, 49]. The bHLH, a superfamily of transcriptome factors (TFs), has been demonstrated to display different biological functions in development of plants [50]. WD-repeat-containing proteins (WDR) are implicated in a variety of functions ranging from signal transduction and transcription regulation to cell cycle control, autophagy and apoptosis [51]. .

We identified 97 MYB, 84 bHLH and 39 WD40 unigenes. Transcript analysis showed that 20 MYB, 12 bHLH and 1 WD40 unigenes were up-regulated and 3MYB, 2 bHLH and 3 WD40 unigenes were down-regulated in branch *vs* leaf. Moreover, 10 MYB, 5 b HLH unigenes were up-regulated and 4 MYB, 2 bHLH and 3 WD40 unigenes were down-regulated in capsule *vs* leaf. Finally, 8 MYB, 4 bHLH and 1 WD40 unigenes were up-regulated and 15 MYB, 12 bHLH unigenes were down-regulated in capsule *vs* branch (Additional file 4). These different expression profile of transcription factors might be responsible for regulating flavonoid biosynthesis and transport in *E. konishii* .

### Phylogenetic tree analysis of key genes involved in flavonoid biosynthesis

Phylogenetic tree analysis of key genes involved in flavonoid biosynthesis was carried using MEGA and the neighbor joining method with 1000 bootstrap replicates, and the results were shown in Additional file 5. The results demonstrated that all the genes involved in flavonoid biosynthesis are homologous gene of one or more known genes.

### Quantitative real-time PCR (qRT-PCR) validationof DEGs involved in flavonoid biosynthesis

**Fig. 7:**
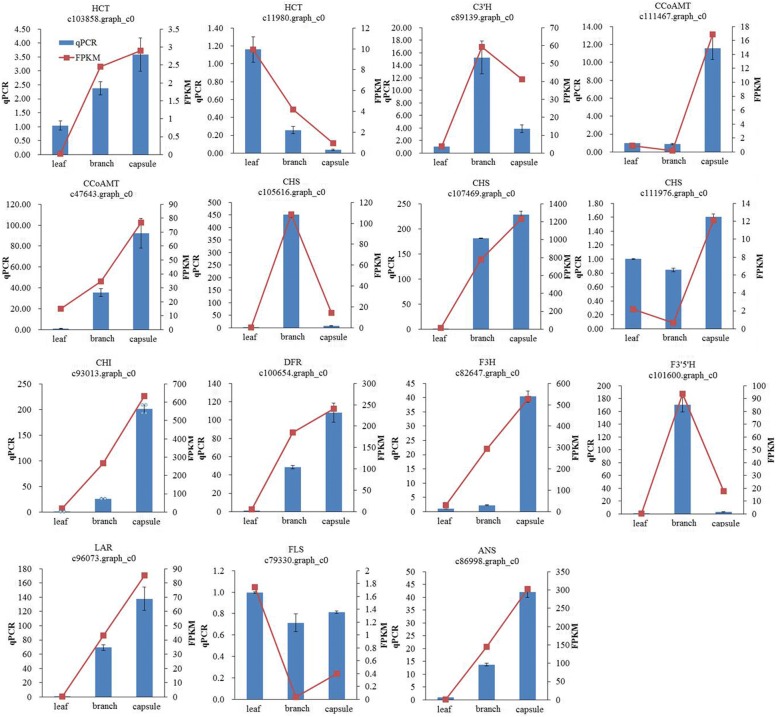
RNA-seq data validation by qRT-PCR. The histograms show the qPCR results of 15 unigenes involved in flavonoid biosynthesis among tissues of *E. konishii*, the red line charts show the FPKM values of these unigenes, and blue bars show the qPCR results, represent the mean ± SD of three biological replicates. The left Y-axis indicates the relative expression levels calculated by qPCR and the right Y-axis indicates the FPKM values of RNA-seq data

To further analyze the consistency of RNA-seq data in this study, a total of 15 genes involved in flavonoid biosynthesis were selected for qRT-PCR validation. The details of those unigenes and primer pairs used in this study are shown in Additional file 6. A comparative analysis of all the selected genes showed a similar expression pattern in qRT-PCR analysis as observed in RNAseq data (Fig. 7), suggesting the reliability in results.

## Discussion

### Rutin accumulation in different organs of *E. konishii*

We isolated a many kinds flavonoids in the previous studies, and the rutin is the most abundant flavonoid in *E. konishii,* which has a long history of use in nutritional supplements for its action against oxidative stress, inflammation, and hyperglycemia [52]. HPLC method was carried in this study to mesure rutin content in different organs (Leaf, Branch and Capsule) of *E. konishii*. Distinctively, the leaf had significantly higher content of rutin compared with other organs, which might signify the essential role of rutin in plant development and physiology.

### Illumina sequencing and sequence annotation

*E. konishii* is a very important traditonal Chinese mdicine. Even though flavoniods are one of the most important active constituents of *E. konishii*, little is known about the mechanisms responsible for flavonoid biosynthesis and metabolism. The aim of this study were to generate a large amount of cDNA sequence data that would facilitate more detailed studies in *E. konishii*, and to identify the genes related to flavonoid biosynthesis and accumulation. In this study, a total of 85,342 unigenes were generated by Illumna sequencing from leaf, branch and capsule of *E. konishii*, and 40,218 (47.13% of 85,342) unigenes provided a significant Blast result. This imformation can provide as equate resources to sthudy *E. konishii* and other related species. But, The annotation percentage of unigenes assembled in this study is lower than other studies, such as *Erigeron breviscapus* (61.7%) [53] and *Hypericum perforatum* (68.86%) [54], this may be due to the length and integrity of the sequences obtained by transcriptome splicing or the lack of genomic information for *E. konishii* and its related species, and it needed to be further study in the future.

### DEGs may play crucial roles in organ function and morphogenesis in *E. konishii*

Higher plants comprise several organs made up of various tissues and cell types. Gene expression patterns differed among the organs of *E. konishii* in our study. Both DEGS were subjected to GO and KEGG annotation, the results promoted our understanding of the genes expression patterns among organs. Most DEGs annotated in basic functions such as “metabolic process”, “cellular process”, “single-organism process”, “organic substance metabolic process”, “primary metabolic process”. But, the DEGs assigned into different KEGG pateways due to the organ-specific manner, such as in leaf, DEGs were significantly enriched in pathways of “photosynthesis, phenylalanine metabolism”, “photosynthesis- antenna proteins” and “carotenoid biosynthesis”. and in branch, DEGs were higher enriched function of “plant hormone signal transduction”, “diterpenoid biosynthesis” and “phenylalanine metabolism”. A functional analysis using the GO and KEGG classification system of DEGs can provide a new insight for studying orgam-specific processes, functions and pathways among different *E. konishii* tissues.

### Candidate genes involved in flavonoid biosynthesis

The relative expression level of candidate genes involved in flavonoids biosynthesis was studied by qRT-PCR, and the results were shown in Fig. 7. As the results showed, the candidate genes involved in flavonoids represent different expression patterns in different organs of *E. konishii*. CHS (c107469.graph_c0), CHI (c93013.graph_c0), DFR (c100654.graph_c0), F3H (c82647.graph_c0), LAR (c96073.graph_c0) and ANS (c86998.graph_c0) showed the same expression pattern in different organs, were all up-regulated in capsule than leaf and branch, and our previous study showed that there were a large amount of anthocyanins in the capsule of *E. konishii*, and the total content of anthocyanins reached 3.880 mg·g^− 1^ [55]. It suggests that the above-mentioned up-regulated genes in capsule may play important roles in the biosynthesis of anthocyanins in capsule of *E. konishii*. We measured the content of rutin in three organs of *E. konishii* by HPLC method, it was significant difference among organs, and in leaf was highest, reached 26.31 ± 2.43 mg·g^− 1^, in branch was lowest (1.43 ± 0.16 mg·g^− 1^), and also the expression level of FLS (c79330.graph_c0), a key gene involved in flavonols biosynthesis, was up-regulated in leaf compared with branch and capsule, and up-regulated in capsule compared with branch, this is consistent with the content of rutin, a flavonol glycoside. These findings suggest that the FLS (c79330.graph_c0) gene may be responsible for the organ-specific biosynthesis of rutin in *E. konishii*.

It has been reported that MYB-bHLH-WD40 complexes can regulate flavonoid biosynthesis pathway through an intricate network [56, 57]. An BoMYB2 together with various BobHLHs from *Brassica oleracea* L. var. botrytis specifically regulated the late anthocyanin biosynthetic pathway genes for anthocyanin biosynthesis [58]. An R2R3-MYB transcription factor, TaMYB14 from *Trifolium arvense* activate proanthocyanidin biosynthesis [59]. An R2R3-MYB transcription factor VvMYBF1 isolated from *Vitis vinifera* ‘Shiraz’ to be a specific activator of flavonol synthase1 (FLS1). We identified 97 MYB unigenes, and 7 MYB (Additional file 4) unigenes were up-regulated in capsule compared with leaf and branch, which suggested that this MYB genes may be responsible for anthocyanin biosynthesis in capsule of *E. konishii*, and another MYB gene (c47321_c0) showed the consistently expression pattern with FLS gene (c79330.graph_c0), it may play important role in regulate the organ-specific biosynthesis of rutin of *E. konishii*.

## Conclusion

In this study, the comparative transcriptome analysis of leaf, branch and capsule of *E. konishii* was performed. We identified putative transcripts involved in biosynthesis and accumulation of flavonoids, and their different expression pattern in the leaf, branch and capsule tissues of *E. konishii* was calculated by DESeq method, which suggests tissues-specific biosynthesis, accumulation and modification of flavonoids might occur in different tissues. This study will contribute significantly to further molecular research of *E. konishii* and other related species.

## Materials and methods

### Plant materials

**Fig. 8:**
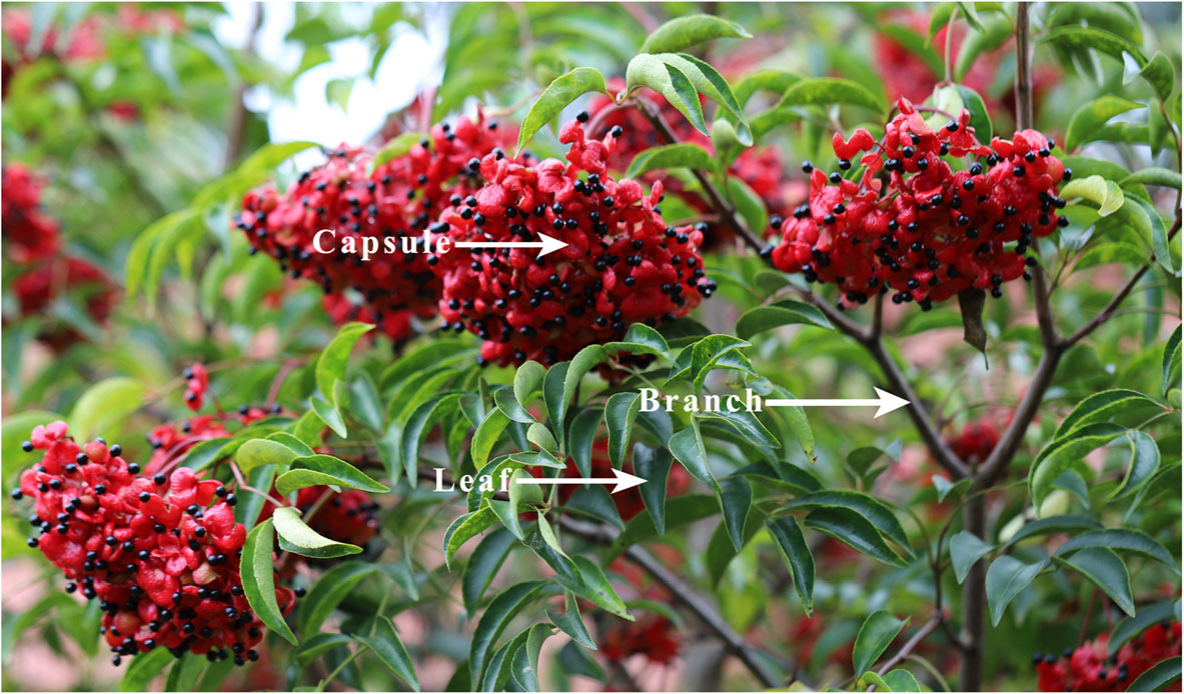
The three organs of *E. konishii* used in this study. This picture is captured by the first author of this manuscript

One year old leaf, branch and capsule of *E. konishii* (Fig. 8) were collected from Fujian Agriculture and Forestry University, Fujian Province, China during November 15th 2016. All samples were harvested, washed and surface dried and then frozen in liquid nitrogen immediately and stored at − 80 °C until required for further processing. Three biological replicates of each organ were used for RNA extraction and transcriptome sequencing.

### Reagends and standards

Methanol of HPLC grade was from Merck (Darmstadt, Germany), other reagents were of analytical grade, rutin (purity>98%) was isolated in our laboratory, and its purity and structure was confirmed by HPLC and by comparison of spectral data to published in the literature.

### Quantitative analysis of Rutin in *E. konishii*

#### Instrumentation and chromatographic conditions

HPLC-DAD was carried out by an Waters HPLC-DAD system comprising a vacuum degasser, binarypump, autosampler, thermostated column compartment, and DAD (Waters, USA), which was used for acquiring chromatograms and UV spectra. An Alltima C18 column (5 μm; 4.6 × 400 mm) was used for HPLC analysis. The mobile phase consisted of 0.1% acetic acid in water (A) and methanol (B), and the procedure was performed with a gradient program of 10–16% (B) at 0–30 min, 16–16% (B) at 30–40 min, 16–30% (B) at 40–80 min, and 30–60% (B) at 80–150 min. The flow rate was 1 mL/min. The detection wavelength was set at 254 nm. The column temperature was set at 30 °C. The injection volume was 10.0 μL.

#### Preparation of standard and sample solutions

Standard solutions were prepared at a concentration of 1 mg/mL with HPLC grade methanol. About 5.0 g of each dried sample was ground into powder and extracted with 10 mL of 70% methanol at 80 °C, for two times, each for an hour. The solvent was condensed in vacuum to yield a crude extract, and the crude extract was dissolved by methanol and made up to 50 mL.

#### RNA extraction and library preparation for transcriptome sequencing

Total RNA was extracted using the Tiangen reagent kit (DP441). A total amount of 3 μg RNA per sample was used as input material for the RNA sample preparations. Sequencing libraries were generated using NEBNext®Ultra™ RNA Library Prep Kit for Illumina® (NEB, USA) following manufacturer’s protocol. Briefly, mRNA was purified from total RNA using poly-T oligo-attached magnetic beads. Fragmentation was carried out using divalent cations under elevated temperature in NEBNext First Strand Synthesis Reaction Buffer (5X). First strand cDNA was synthesized using random hexamer primer and M-MuLV Reverse Transcriptase (RNase H-). Second strand cDNA synthesis was subsequently performed using DNA Polymerase I and RNase H. Remaining overhangs were converted into blunt ends via exonuclease/polymerase activities. After adenylation of 3′ ends, NEBNext Adaptor with hairpin loop structure were ligated to prepare for hybridization. In order to select cDNA fragments of preferentially 150~200 bp in length, the library fragments were purified with AMPure XP system (Beckman Coulter, Beverly, USA). 3 μL USER Enzyme (NEB, USA) was used with size-selected, adaptor-ligated cDNA at 37 °C for 15 min followed by 5 min at 95 °C before PCR. PCR was performed with Phusion High-Fidelity DNA polymerase, Universal PCR primers and Index (X) Primer. At last, PCR products were purified (AMPure XP system) and library quality was assessed on the Agilent Bioanalyzer 2100 system.

#### Clustering and sequencing

The clustering of the index-coded samples was performed on a cBot Cluster Generation System using TruSeq PE Cluster Kit v3-cBot-HS (Illumia) according to the manufacturer’s instructions. After cluster generation, the library preparations were sequenced on an Illumina Hiseq 2000 platform and paired-end reads were generated.

#### Quality control

Raw data (raw reads) of fastq format were firstly processed through in-house perl scripts. In this step, clean data (clean reads) were obtained by removing reads containing adapter, reads containing ploy-N and low quality reads from raw data. At the same time, Q20, Q30, GC-content and sequence duplication level of the clean data were calculated. All the downstream analyses were based on clean data with high quality.

#### Transcriptome assembly

The left files (read1 files) from all libraries were pooled into one big left.fq file, and right files (read2 files) into one big right.fq file. Transcriptome assembly was accomplished based on the left.fq and right.fq using Trinity [60] with min_kmer_cov set to 2 by default and all other parameters set default.

#### Gene functional annotation

Gene function was annotated based on the following databases: NR (NCBI non-redundant protein sequences), KOG/COG/eggNOG (Clusters of Orthologous Groups of proteins), Swiss-Prot (A manually annotated and reviewed protein sequence database), KEGG (Kyoto Encyclopedia of Genes and Genomes), and GO (Gene Ontology).

#### Quantification of gene expression levels

Gene expression levels were estimated by RSEM [61] for each sample: 1) clean data were mapped back onto the assembled transcriptome and 2) read-counts for each gene was obtained from the mapping results.

#### Differential expression analysis

Differential expression analysis of two conditions/groups was performed using the DESeq R package. DESeq provide statistical procedures to determine differential expression in digital gene expression data using a model based on the negative binomial distribution. The resulting *P* values were adjusted using the Benjamini and Hochberg’s approach for controlling the false discovery rate (FDR). Genes with an adjusted *P*-value < 0.05 found by DESeq were assigned as differentially expressed.

#### GO enrichment analysis

Gene Ontology (GO) enrichment analysis of the differentially expressed genes (DEGs) was implemented using topGO R packages based Kolmogorov–Smirnov test.

#### KEGG pathway enrichment analysis

KEGG [62] is a database resource for understanding high-level functions and utilities of the biological system, such as the cell, the organism and the ecosystem, from molecular-level information, especially large-scale molecular datasets generated by genome sequencing and other high-throughput experimental technologies (http://www.genome.jp/kegg/). We used KOBAS software [63] to test the statistical enrichment of differential expression genes in KEGG pathways.

#### Quantitative PCR (qRT-PCR) analysis

Fifteen differential expressed genes (c103858.graph_c0, c111980.graph_c0, c89139.graph_c0, c111467.graph_c0, c47643.graph_c0, c105616.graph_c0, c107469.graph_c0, c111976.graph_c0, c93013.graph_c0, c100654.graph_c0, c82647.graph_c0, c101600.graph_c0, c96073.graph_c0, c79330.graph_c0, c86998.graph_c0) involved flavonoid biosynthesis were selected for quantitive real-time PCR (qRT-PCR), the primers of all selected genes were designed by Primer Premier 5 (Additional file 6). The qRT-PCR analysis of each gene was performed on a 7500 Fast ABI Real-time PCR system (Applied Bio, US) using FastStart Universial SYBR Green Master (ROCHE, Switzerland). A 20 uL reaction mixture contain 10 uL 2 × SYBR Green Master, 0.4 uL forward primer (10 uM), 0.4 uL reverse primer (10 uM), 2 uL cDNA and 7.2 uLdd H_2_O in a 96-well plates. The amplification conditions were as follows: 50 °C for 2 min, 95 °C for 10 min; 40 cycles of 95 °C for 15 s and 60 °C for 30s. Relative gene expression levels were calculated by 2^-△△Ct^, *GADPH* and *GSTU1* were selected as internal reference genes according to previous studies [64].

#### Phylogenetic analysis

The phylogenetic analysis based on the amino acid sequences was performed using MEGA (version 7.0, the laboratory at the Pennsylvania State University, St Collie, PA, USA) and the neighbor joining method with 1000 bootstrap replicates.

## Additional files

Additional file 1: Total unigenes assigned to 128 KEGG pathways (DOCX 23 kb)

Additional file 2: GO annotation of DEGs (DOCX 315 kb)

Additional file 3: Significantly enriched KEGG pathways in DEGs between different tissues in *E. konishii* Hayata (DOCX 17 kb)

Additional file 4: Candidate genes involved in Flavonoid accumulation (DOCX 38 kb)

Additional file 5: Phylogenetic tree analysis of Candidate genes involved in Flavonoid biosynthesis (DOCX 15761 kb)

Additional file 6: Primers used in validation experiment of gene expression by qRT-PCR (DOCX 13 kb)

## Abbreviations

4CL: 4-coumarate CoA ligase
ANR: Anthocyanidinreductase
ANS: Leucoanthocyanidin dioxygenase
C3’H: Coumaroylquinate 3′-monooxygenase
C4H: Cinnamate 4-hydroxylase
CCoAMT: Caffeoyl-CoA O-methyltransferase
CHI: Chalcone isomerase
CHS: Chalcone synthase
CYP450: Cytochrome P450.
DFR: Dihydroflavonol 4-reductase
F3’5’H: Flavonoid 3′,5′-hydroxylase
F3’H: Flavonoid 3′-hydroxylase
F3H: Flavanone 3-hydroxylase
FLS: Flavonolsynthese
HCT: Shikimate O-hydroxycinnamoyltransferase
LAR: Leucoanthocyanidin reductase
OMT: O-methyltransferase
PAL: Phenylalanine ammonia lyase
UGT: UDP-glycosyltransferase
