# Supplementary material for: Comparative transcriptome among Euscaphis konishii Hayata tissues and analysis of genes involved in flavonoid biosynthesis and accumulation
Source: BMC Genomics. 2019 Jan 9;20:24. doi: 10.1186/s12864-018-5354-x (PMC6327468; doi:10.1186/s12864-018-5354-x)
Supplement: Supplementary file 1 — Total unigenes assigned to 128 KEGG pathways (DOCX 23 kb) [file 12864_2018_5354_MOESM1_ESM.docx]

**Table S1. Total unigenes assigned to 128 KEGG pathways**

| #pathway | pathway_id | Gene_number |
| --- | --- | --- |
| Glycolysis / Gluconeogenesis | ko00010 | 325 |
| Citrate cycle (TCA cycle) | ko00020 | 187 |
| Pentose phosphate pathway | ko00030 | 140 |
| Pentose and glucuronate interconversions | ko00040 | 160 |
| Fructose and mannose metabolism | ko00051 | 117 |
| Galactose metabolism | ko00052 | 123 |
| Ascorbate and aldarate metabolism | ko00053 | 115 |
| Fatty acid biosynthesis | ko00061 | 99 |
| Fatty acid elongation | ko00062 | 40 |
| Fatty acid degradation | ko00071 | 143 |
| Synthesis and degradation of ketone bodies | ko00072 | 16 |
| Cutin, suberine and wax biosynthesis | ko00073 | 29 |
| Steroid biosynthesis | ko00100 | 62 |
| Ubiquinone and other terpenoid-quinone biosynthesis | ko00130 | 52 |
| Oxidative phosphorylation | ko00190 | 336 |
| Photosynthesis | ko00195 | 136 |
| Photosynthesis - antenna proteins | ko00196 | 76 |
| Purine metabolism | ko00230 | 251 |
| Caffeine metabolism | ko00232 | 3 |
| Pyrimidine metabolism | ko00240 | 169 |
| Alanine, aspartate and glutamate metabolism | ko00250 | 139 |
| Glycine, serine and threonine metabolism | ko00260 | 145 |
| Cysteine and methionine metabolism | ko00270 | 211 |
| Valine, leucine and isoleucine degradation | ko00280 | 145 |
| Valine, leucine and isoleucine biosynthesis | ko00290 | 63 |
| Lysine biosynthesis | ko00300 | 24 |
| Lysine degradation | ko00310 | 106 |
| Arginine and proline metabolism | ko00330 | 183 |
| Histidine metabolism | ko00340 | 59 |
| Tyrosine metabolism | ko00350 | 75 |
| Phenylalanine metabolism | ko00360 | 132 |
| Tryptophan metabolism | ko00380 | 129 |
| Phenylalanine, tyrosine and tryptophan biosynthesis | ko00400 | 77 |
| beta-Alanine metabolism | ko00410 | 123 |
| Taurine and hypotaurine metabolism | ko00430 | 39 |
| Selenocompound metabolism | ko00450 | 47 |
| Cyanoamino acid metabolism | ko00460 | 93 |
| Glutathione metabolism | ko00480 | 202 |
| Starch and sucrose metabolism | ko00500 | 297 |
| N-Glycan biosynthesis | ko00510 | 70 |
| Other glycan degradation | ko00511 | 27 |
| Other types of O-glycan biosynthesis | ko00514 | 13 |
| Amino sugar and nucleotide sugar metabolism | ko00520 | 195 |
| Glycosaminoglycan degradation | ko00531 | 14 |
| Glycerolipid metabolism | ko00561 | 138 |
| Inositol phosphate metabolism | ko00562 | 119 |
| Glycosylphosphatidylinositol(GPI)-anchor biosynthesis | ko00563 | 24 |
| Glycerophospholipid metabolism | ko00564 | 162 |
| Ether lipid metabolism | ko00565 | 59 |
| Arachidonic acid metabolism | ko00590 | 48 |
| Linoleic acid metabolism | ko00591 | 46 |
| alpha-Linolenic acid metabolism | ko00592 | 131 |
| Sphingolipid metabolism | ko00600 | 72 |
| Glycosphingolipid biosynthesis - globo series | ko00603 | 16 |
| Glycosphingolipid biosynthesis - ganglio series | ko00604 | 8 |
| Pyruvate metabolism | ko00620 | 268 |
| Glyoxylate and dicarboxylate metabolism | ko00630 | 239 |
| Propanoate metabolism | ko00640 | 86 |
| Butanoate metabolism | ko00650 | 59 |
| C5-Branched dibasic acid metabolism | ko00660 | 22 |
| One carbon pool by folate | ko00670 | 41 |
| Carbon fixation in photosynthetic organisms | ko00710 | 222 |
| Thiamine metabolism | ko00730 | 43 |
| Riboflavin metabolism | ko00740 | 10 |
| Vitamin B6 metabolism | ko00750 | 23 |
| Nicotinate and nicotinamide metabolism | ko00760 | 25 |
| Pantothenate and CoA biosynthesis | ko00770 | 50 |
| Biotin metabolism | ko00780 | 30 |
| Lipoic acid metabolism | ko00785 | 10 |
| Folate biosynthesis | ko00790 | 25 |
| Porphyrin and chlorophyll metabolism | ko00860 | 77 |
| Terpenoid backbone biosynthesis | ko00900 | 92 |
| Monoterpenoid biosynthesis | ko00902 | 7 |
| Limonene and pinene degradation | ko00903 | 33 |
| Diterpenoid biosynthesis | ko00904 | 31 |
| Brassinosteroid biosynthesis | ko00905 | 19 |
| Carotenoid biosynthesis | ko00906 | 49 |
| Zeatin biosynthesis | ko00908 | 18 |
| Sesquiterpenoid and triterpenoid biosynthesis | ko00909 | 30 |
| Nitrogen metabolism | ko00910 | 79 |
| Sulfur metabolism | ko00920 | 75 |
| Phenylpropanoid biosynthesis | ko00940 | 196 |
| Flavonoid biosynthesis | ko00941 | 46 |
| Anthocyanin biosynthesis | ko00942 | 4 |
| Isoflavonoid biosynthesis | ko00943 | 3 |
| Flavone and flavonol biosynthesis | ko00944 | 6 |
| Stilbenoid, diarylheptanoid and gingerol biosynthesis | ko00945 | 24 |
| Isoquinoline alkaloid biosynthesis | ko00950 | 40 |
| Tropane, piperidine and pyridine alkaloid biosynthesis | ko00960 | 49 |
| Betalain biosynthesis | ko00965 | 2 |
| Glucosinolate biosynthesis | ko00966 | 5 |
| Aminoacyl-tRNA biosynthesis | ko00970 | 102 |
| Biosynthesis of unsaturated fatty acids | ko01040 | 110 |
| Carbon metabolism | ko01200 | 664 |
| 2-Oxocarboxylic acid metabolism | ko01210 | 160 |
| Fatty acid metabolism | ko01212 | 211 |
| Degradation of aromatic compounds | ko01220 | 29 |
| Biosynthesis of amino acids | ko01230 | 561 |
| Vancomycin resistance | ko01502 | 2 |
| ABC transporters | ko02010 | 74 |
| Ribosome biogenesis in eukaryotes | ko03008 | 158 |
| Ribosome | ko03010 | 856 |
| RNA transport | ko03013 | 333 |
| mRNA surveillance pathway | ko03015 | 203 |
| RNA degradation | ko03018 | 199 |
| RNA polymerase | ko03020 | 53 |
| Basal transcription factors | ko03022 | 55 |
| DNA replication | ko03030 | 88 |
| Spliceosome | ko03040 | 327 |
| Proteasome | ko03050 | 130 |
| Protein export | ko03060 | 79 |
| Base excision repair | ko03410 | 61 |
| Nucleotide excision repair | ko03420 | 84 |
| Mismatch repair | ko03430 | 53 |
| Homologous recombination | ko03440 | 62 |
| Non-homologous end-joining | ko03450 | 7 |
| Phosphatidylinositol signaling system | ko04070 | 99 |
| Plant hormone signal transduction | ko04075 | 407 |
| Ubiquitin mediated proteolysis | ko04120 | 238 |
| Sulfur relay system | ko04122 | 27 |
| SNARE interactions in vesicular transport | ko04130 | 51 |
| Regulation of autophagy | ko04140 | 58 |
| Protein processing in endoplasmic reticulum | ko04141 | 455 |
| Endocytosis | ko04144 | 270 |
| Phagosome | ko04145 | 193 |
| Peroxisome | ko04146 | 237 |
| Plant-pathogen interaction | ko04626 | 292 |
| Circadian rhythm - plant | ko04712 | 84 |
