# Supplementary material for: Comparative transcriptome among Euscaphis konishii Hayata tissues and analysis of genes involved in flavonoid biosynthesis and accumulation
Source: BMC Genomics. 2019 Jan 9;20:24. doi: 10.1186/s12864-018-5354-x (PMC6327468; doi:10.1186/s12864-018-5354-x)
Supplement: Supplementary file 2 — GO annotation of DEGs (DOCX 315 kb) [file 12864_2018_5354_MOESM2_ESM.docx]

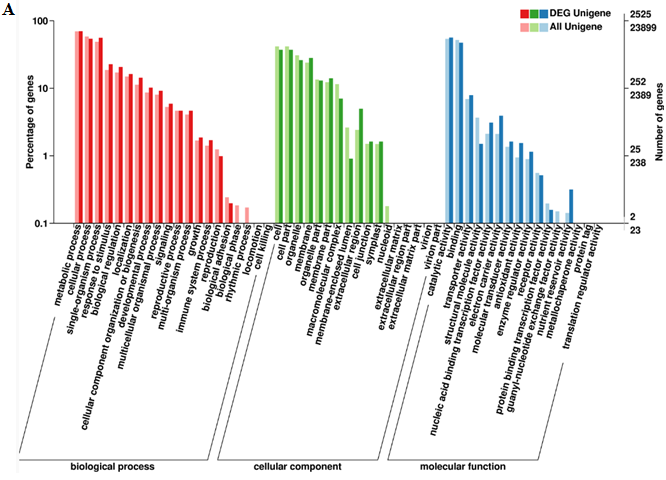

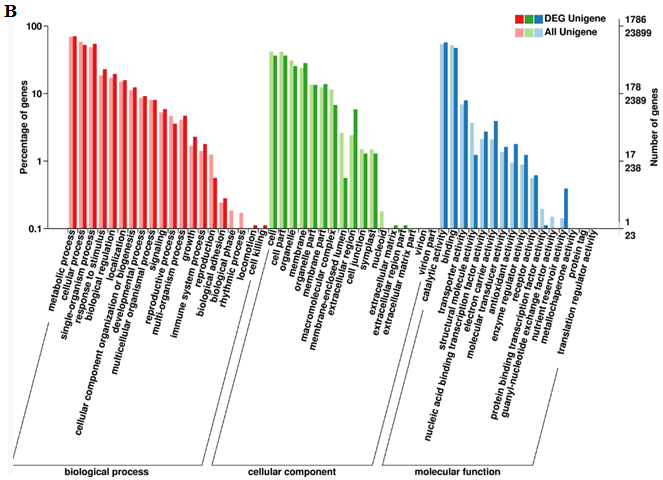


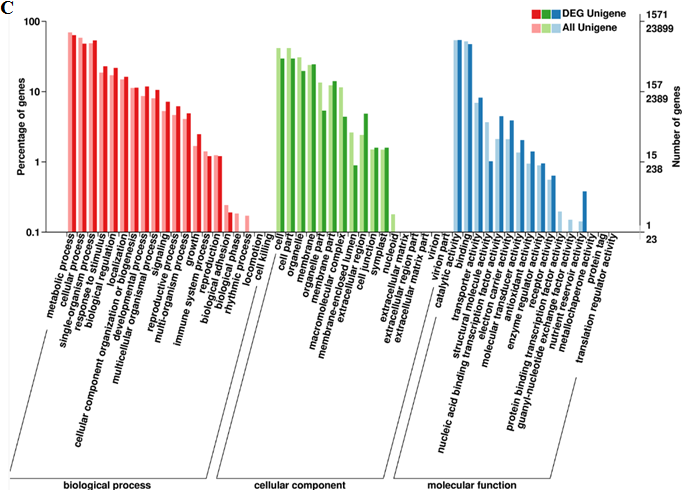


**Figure S1. GO annotation of DEGs.** (A) Leaf vs Branch, (B) Leaf vs Capsule, (C) Branch vs Capsule.Unigenes were annotated in three categories: cellular components, molecular functions, and biological process. Right y-axis indicates the number of genes in a category; left y-axis indicates the genes percentage in a speciﬁc. The light color indicates all unigenes, dark color indicates DEG unigenes.
