# Supplementary material for: Comparative transcriptome among Euscaphis konishii Hayata tissues and analysis of genes involved in flavonoid biosynthesis and accumulation
Source: BMC Genomics. 2019 Jan 9;20:24. doi: 10.1186/s12864-018-5354-x (PMC6327468; doi:10.1186/s12864-018-5354-x)
Supplement: Supplementary file 3 — Significantly enriched KEGG pathways in DEGs between different tissues in E. konishii Hayata (DOCX 17 kb) [file 12864_2018_5354_MOESM3_ESM.docx]

**Table S2. Significantly enriched KEGG pathways in DEGs between different tissues in *Euscaphis konishii* Hayata**

| **#Pathway** | **ko_ID** | **DEG_in_Pathway** | **AllGene_in_Pathway** | **P-value** | **Corrected_P-value** |
| --- | --- | --- | --- | --- | --- |
| **Leaf vs Branch** |  |  |  |  |  |
| Phenylpropanoid biosynthesis | ko00940 | 45 | 196 | 8.38E-11 | 9.14E-09 |
| Cyanoamino acid metabolism | ko00460 | 28 | 93 | 4.97E-10 | 5.42E-08 |
| Carotenoid biosynthesis | ko00906 | 19 | 49 | 2.77E-09 | 3.02E-07 |
| Starch and sucrose metabolism | ko00500 | 55 | 297 | 3.78E-09 | 4.12E-07 |
| Plant hormone signal transduction | ko04075 | 66 | 407 | 2.70E-08 | 2.95E-06 |
| Photosynthesis | ko00195 | 31 | 136 | 9.15E-08 | 9.98E-06 |
| Flavonoid biosynthesis | ko00941 | 15 | 46 | 1.79E-06 | 0.000194838 |
| Phenylalanine metabolism | ko00360 | 27 | 132 | 5.70E-06 | 0.000621769 |
| Taurine and hypotaurine metabolism | ko00430 | 12 | 39 | 3.77E-05 | 0.004104235 |
| Diterpenoid biosynthesis | ko00904 | 10 | 31 | 0.00010751 | 0.011718577 |
| Vitamin B6 metabolism | ko00750 | 8 | 23 | 0.000296234 | 0.032289453 |
| Arachidonic acid metabolism | ko00590 | 12 | 48 | 0.000339905 | 0.037049637 |
| **Leaf vs Capsule** |  |  |  |  |  |
| **#Pathway** | **ko_ID** | **DEG_in_Pathway** | **AllGene_in_Pathway** | **P-value** | **Corrected_P-value** |
| Photosynthesis | ko00195 | 37 | 136 | 7.82E-13 | 8.45E-11 |
| Phenylalanine metabolism | ko00360 | 32 | 132 | 2.96E-11 | 3.19E-09 |
| Phenylpropanoid biosynthesis | ko00940 | 40 | 196 | 3.26E-11 | 3.52E-09 |
| Cyanoamino acid metabolism | ko00460 | 23 | 93 | 1.21E-08 | 1.31E-06 |
| Flavonoid biosynthesis | ko00941 | 15 | 46 | 8.27E-08 | 8.94E-06 |
| Photosynthesis - antenna proteins | ko00196 | 19 | 76 | 1.90E-07 | 2.05E-05 |
| Carotenoid biosynthesis | ko00906 | 14 | 49 | 1.38E-06 | 0.000149 |
| Pentose and glucuronate interconversions | ko00040 | 27 | 160 | 2.91E-06 | 0.000315 |
| Diterpenoid biosynthesis | ko00904 | 10 | 31 | 1.39E-05 | 0.001501 |
| Glycine, serine and threonine metabolism | ko00260 | 24 | 145 | 1.45E-05 | 0.001567 |
| Taurine and hypotaurine metabolism | ko00430 | 11 | 39 | 2.17E-05 | 0.002343 |
| Arachidonic acid metabolism | ko00590 | 12 | 48 | 3.50E-05 | 0.003776 |
| Tropane, piperidine and pyridine alkaloid biosynthesis | ko00960 | 12 | 49 | 4.36E-05 | 0.004714 |
| Starch and sucrose metabolism | ko00500 | 36 | 297 | 0.000148 | 0.015944 |
| Isoquinoline alkaloid biosynthesis | ko00950 | 10 | 40 | 0.000157 | 0.01698 |
| Ubiquinone and other terpenoid-quinone biosynthesis | ko00130 | 11 | 52 | 0.000365 | 0.039458 |
| Carbon fixation in photosynthetic organisms | ko00710 | 28 | 222 | 0.000417 | 0.045059 |
| **Branch vs Capsule** |  |  |  |  |  |
| **#Pathway** | **ko_ID** | **DEG_in_Pathway** | **AllGene_in_Pathway** | **P-value** | **Corrected_P-value** |
| Plant hormone signal transduction | ko04075 | 61 | 407 | 1.23E-12 | 1.32E-10 |
| Diterpenoid biosynthesis | ko00904 | 12 | 31 | 5.36E-09 | 5.73E-07 |
| Phenylalanine metabolism | ko00360 | 19 | 132 | 9.82E-06 | 0.001050289 |
| Starch and sucrose metabolism | ko00500 | 31 | 297 | 1.80E-05 | 0.001927555 |
| Taurine and hypotaurine metabolism | ko00430 | 9 | 39 | 5.58E-05 | 0.005973004 |
| Carotenoid biosynthesis | ko00906 | 10 | 49 | 6.68E-05 | 0.007150963 |
| Cyanoamino acid metabolism | ko00460 | 14 | 93 | 8.86E-05 | 0.009475411 |
| Isoflavonoid biosynthesis | ko00943 | 3 | 3 | 9.92E-05 | 0.010615222 |
| Phenylpropanoid biosynthesis | ko00940 | 22 | 196 | 0.000105 | 0.011191912 |
| Galactose metabolism | ko00052 | 16 | 123 | 0.000169 | 0.018079413 |
| Pentose and glucuronate interconversions | ko00040 | 18 | 160 | 0.000428 | 0.045761235 |
