# Supplementary material for: Comparative transcriptome among Euscaphis konishii Hayata tissues and analysis of genes involved in flavonoid biosynthesis and accumulation
Source: BMC Genomics. 2019 Jan 9;20:24. doi: 10.1186/s12864-018-5354-x (PMC6327468; doi:10.1186/s12864-018-5354-x)
Supplement: Supplementary file 4 — Candidate genes involved in Flavonoid accumulation (DOCX 38 kb) [file 12864_2018_5354_MOESM4_ESM.docx]

**Table S3. Candidate genes involved in Flavonoid accumulation**

| #ID |  | | Function annotation |  | FPKM |  |
| --- | --- | --- | --- | --- | --- | --- |
|  |  | |  | Leaf | Branch | Capsule |
| **Structural genes** | | | | | | |
| c101429.graph_c0 | | Phenylalanine ammonia lyse (PAL) | | 139.0679 | 76.8753 | 48.8899 |
| c108615.graph_c0 | | Phenylalanine ammonia lyse (PAL) | | 0.8286 | 41.4556 | 16.2495 |
| c108778.graph_c0 | | Phenylalanine ammonia lyse (PAL) | | 13.8403 | 29.5891 | 11.4303 |
| c108807.graph_c0 | | Phenylalanine ammonia lyse (PAL) | | 7.3697 | 13.8338 | 4.9720 |
| c109138.graph_c0 | | Phenylalanine ammonia lyse (PAL) | | 9.7796 | 22.7991 | 8.0757 |
| c117934.graph_c0 | | Phenylalanine ammonia lyse (PAL) | | 0.1245 | 0.3246 | 0.0746 |
| c122971.graph_c0 | | Phenylalanine ammonia lyse (PAL) | | 0.1397 | 0.0542 | 0.0558 |
| c1461.graph_c0 | | Phenylalanine ammonia lyse (PAL) | | 0.2597 | 0.2701 | 0.1576 |
| c23947.graph_c0 | | Phenylalanine ammonia lyse (PAL) | | 5.2470 | 13.6516 | 4.8788 |
| c26856.graph_c0 | | Phenylalanine ammonia lyse (PAL) | | 4.3944 | 20.2729 | 5.5988 |
| c58422.graph_c0 | | Phenylalanine ammonia lyse (PAL) | | 0.1394 | 0.1343 | 0.1800 |
| c97411.graph_c0 | | Phenylalanine ammonia lyse (PAL) | | 6.7122 | 57.8665 | 93.2861 |
| c114061.graph_c0 | | Cinnamate-4-hydroxylase (C4H) | | 0.1602 | 5.3814 | 0.1952 |
| c29912.graph_c0 | | Cinnamate-4-hydroxylase (C4H) | | 0.2403 | 0.3105 | 0.3426 |
| c31496.graph_c0 | | Cinnamate-4-hydroxylase (C4H) | | 0.0000 | 4.1970 | 0.3356 |
| c94660.graph_c0 | | Cinnamate-4-hydroxylase (C4H) | | 0.8087 | 1.5232 | 0.6469 |
| c102085.graph_c1 | | 4-coumarate CoA ligase (4CL) | | 48.1662 | 47.3406 | 110.9061 |
| c107760.graph_c0 | | 4-coumarate CoA ligase (4CL) | | 29.2583 | 107.3228 | 79.4974 |
| c108137.graph_c0 | | 4-coumarate CoA ligase (4CL) | | 16.2411 | 18.9706 | 18.3501 |
| c108109.graph_c0 | | 4-coumarate CoA ligase (4CL) | | 2.7615 | 68.8392 | 22.5154 |
| c108197.graph_c0 | | 4-coumarate CoA ligase (4CL) | | 1.1336 | 27.6562 | 7.4115 |
| c115342.graph_c0 | | 4-coumarate CoA ligase (4CL) | | 2.4357 | 0.3449 | 0.2516 |
| c140153.graph_c0 | | 4-coumarate CoA ligase (4CL) | | 0.1132 | 0.1793 | 0.0000 |
| c140551.graph_c0 | | 4-coumarate CoA ligase (4CL) | | 0.2627 | 0.1357 | 0.0000 |
| c147444.graph_c0 | | 4-coumarate CoA ligase (4CL) | | 0.0000 | 0.3626 | 0.0000 |
| c15578.graph_c0 | | 4-coumarate CoA ligase (4CL) | | 0.0734 | 0.4403 | 0.0000 |
| c23876.graph_c0 | | 4-coumarate CoA ligase (4CL) | | 3.0445 | 59.2457 | 21.8302 |
| c27315.graph_c0 | | 4-coumarate CoA ligase (4CL) | | 4.5612 | 7.2330 | 11.4575 |
| c54965.graph_c0 | | 4-coumarate CoA ligase (4CL) | | 0.0000 | 0.4163 | 0.0000 |
| c66051.graph_c0 | | 4-coumarate CoA ligase (4CL) | | 0.0634 | 0.1890 | 0.0000 |
| c91976.graph_c0 | | 4-coumarate CoA ligase (4CL) | | 0.2049 | 0.3705 | 0.3321 |
| c103796.graph_c0 | | Shikimate O-hydroxycinnamoyltransferase (HCT) | | 0.0243 | 2.4584 | 2.8931 |
| c104505.graph_c1 | | Shikimate O-hydroxycinnamoyltransferase (HCT) | | 16.7142 | 29.5448 | 53.1654 |
| c111980.graph_c0 | | Shikimate O-hydroxycinnamoyltransferase (HCT) | | 26.5626 | 75.6597 | 45.7185 |
| c126223.graph_c0 | | Shikimate O-hydroxycinnamoyltransferase (HCT) | | 9.9532 | 4.1650 | 0.9321 |
| c134286.graph_c0 | | Shikimate O-hydroxycinnamoyltransferase (HCT) | | 0.0000 | 0.2992 | 0.1867 |
| c43728.graph_c0 | | Shikimate O-hydroxycinnamoyltransferase (HCT) | | 0.1055 | 0.2142 | 0.0622 |
| c78422.graph_c0 | | Shikimate O-hydroxycinnamoyltransferase (HCT) | | 0.0103 | 0.8828 | 0.0000 |
| c78422.graph_c0 | | Shikimate O-hydroxycinnamoyltransferase (HCT) | | 76.7150 | 282.8121 | 106.7213 |
| c73512.graph_c0 | | Coumaroylquinate(coumaroylshikimate)3'-monooxygenase (C3'H) | | 0.1171 | 0.2032 | 0.1156 |
| c89022.graph_c0 | | Coumaroylquinate(coumaroylshikimate)3'-monooxygenase (C3'H) | | 0.0192 | 0.4212 | 0.0000 |
| c89139.graph_c0 | | Coumaroylquinate(coumaroylshikimate)3'-monooxygenase (C3'H) | | 3.8653 | 59.3734 | 41.2210 |
| c109231.graph_c0 | | Caffeoyl-CoA O-methyltransferase (CCoAMT) | | 0.1303 | 1.9285 | 0.5845 |
| c111467.graph_c0 | | Caffeoyl-CoA O-methyltransferase (CCoAMT) | | 0.8740 | 0.1700 | 16.8662 |
| c47643.graph_c0 | | Caffeoyl-CoA O-methyltransferase (CCoAMT) | | 15.0266 | 34.5401 | 76.9265 |
| c94269.graph_c0 | | Caffeoyl-CoA O-methyltransferase (CCoAMT) | | 0.2509 | 0.3124 | 0.2532 |
| c94319.graph_c0 | | Caffeoyl-CoA O-methyltransferase (CCoAMT) | | 24.7381 | 112.8473 | 46.9741 |
| c98251.graph_c0 | | Caffeoyl-CoA O-methyltransferase (CCoAMT) | | 14.3849 | 15.0692 | 18.6002 |
| c105616.graph_c0 | | Chalcone synthase (CHS) | | 0.2737 | 108.7412 | 14.2793 |
| c107469.graph_c0 | | Chalcone synthase (CHS) | | 15.1832 | 775.5909 | 1232.2124 |
| c111976.graph_c0 | | Chalcone synthase (CHS) | | 2.1984 | 0.6522 | 12.0952 |
| c124391.graph_c0 | | Chalcone synthase (CHS) | | 0.1408 | 0.1499 | 0.2602 |
| c125874.graph_c0 | | Chalcone synthase (CHS) | | 0.4365 | 0.0241 | 0.1784 |
| c126165.graph_c0 | | Chalcone synthase (CHS) | | 0.4413 | 0.1061 | 0.0348 |
| c131451.graph_c0 | | Chalcone synthase (CHS) | | 0.0000 | 0.0387 | 0.5313 |
| c21988.graph_c0 | | Chalcone synthase (CHS) | | 0.1867 | 0.4701 | 0.4130 |
| c72240.graph_c0 | | Chalcone synthase (CHS) | | 0.3543 | 0.3319 | 0.8206 |
| c72954.graph_c0 | | Chalcone synthase (CHS) | | 1.9463 | 0.2198 | 0.1951 |
| c81469.graph_c0 | | Chalcone synthase (CHS) | | 0.0000 | 0.1365 | 0.6152 |
| c93013.graph_c0 | | Chalcone isomerase (CHI) | | 19.6338 | 266.0583 | 633.8203 |
| c100654.graph_c0 | | Dihydroflavonol 4-reductase (DFR) | | 5.7512 | 184.8659 | 241.2763 |
| c119223.graph_c0 | | Flavanone-3-hydroxylase (F3H) | | 0.1360 | 0.3885 | 0.7259 |
| c82647.graph_c0 | | Flavanone-3-hydroxylase (F3H) | | 30.3809 | 294.5326 | 528.4220 |
| c101600.graph_c0 | | Flavonoid-3'5'-hydroxylase (F3'5'H) | | 0.2583 | 93.6205 | 17.7807 |
| c37726.graph_c0 | | Flavonoid-3'5'-hydroxylase (F3'5'H) | | 0.0616 | 0.1434 | 0.3134 |
| c94604.graph_c0 | | Flavonoid-3'5'-hydroxylase (F3'5'H) | | 1.4594 | 3.2918 | 1.6688 |
| c98246.graph_c0 | | Flavonoid-3'-hydroxylase (F3'H) | | 104.1473 | 114.7303 | 351.1741 |
| c96073.graph_c0 | | Leucoanthocyanidin reductase (LAR) | | 0.3634 | 43.0477 | 85.1813 |
| c106977.graph_c1 | | Anthocyanidin reductase (ANR) | | 56.1225 | 98.0869 | 48.6885 |
| c75641.graph_c0 | | Anthocyanidin reductase (ANR) | | 0.0647 | 0.1842 | 0.1751 |
| c79330.graph_c0 | | Flaconol synthase (FLS) | | 1.7422 | 0.0357 | 0.4003 |
| c79330.graph_c1 | | Flaconol synthase (FLS) | | 0.2861 | 0.0000 | 0.1752 |
| c86998.graph_c0 | | Anthocyanidin synthase (ANS) | | 0.8637 | 144.1818 | 302.3010 |
| **Modification genes** | |  | |  |  |  |
| c100191.graph_c0 | | UDP-glycosyltransferase (UGT) | | 6.5780 | 6.3810 | 14.2997 |
| c101298.graph_c0 | | UDP-glycosyltransferase (UGT) | | 0.9775 | 1.0337 | 2.8099 |
| c102025.graph_c0 | | UDP-glycosyltransferase (UGT) | | 0.3631 | 3.9200 | 18.4377 |
| c104952.graph_c0 | | UDP-glycosyltransferase (UGT) | | 1.2483 | 1.0618 | 0.9442 |
| c105064.graph_c0 | | UDP-glycosyltransferase (UGT) | | 149.5634 | 44.1865 | 30.6957 |
| c108655.graph_c0 | | UDP-glycosyltransferase (UGT) | | 1.2856 | 9.9660 | 8.4384 |
| c109047.graph_c0 | | UDP-glycosyltransferase (UGT) | | 7.3224 | 2.5949 | 52.6576 |
| c110622.graph_c0 | | UDP-glycosyltransferase (UGT) | | 0.0163 | 0.1306 | 24.4028 |
| c112729.graph_c0 | | UDP-glycosyltransferase (UGT) | | 3.1027 | 0.0782 | 3.4362 |
| c113572.graph_c0 | | UDP-glycosyltransferase (UGT) | | 0.5007 | 1.2623 | 6.5296 |
| c25698.graph_c0 | | UDP-glycosyltransferase (UGT) | | 8.2958 | 3.7050 | 1.9396 |
| c40753.graph_c0 | | UDP-glycosyltransferase (UGT) | | 4.1900 | 0.0829 | 3.3541 |
| c83288.graph_c0 | | UDP-glycosyltransferase (UGT) | | 3.7800 | 12.8579 | 0.0605 |
| c88287.graph_c0 | | UDP-glycosyltransferase (UGT) | | 3.5066 | 34.3637 | 20.0357 |
| c91453.graph_c0 | | UDP-glycosyltransferase (UGT) | | 25.3261 | 14.2959 | 209.7437 |
| c94437.graph_c0 | | UDP-glycosyltransferase (UGT) | | 3.4382 | 30.0477 | 2.2194 |
| c95167.graph_c0 | | UDP-glycosyltransferase (UGT) | | 16.6359 | 1.8713 | 7.8125 |
| c96495.graph_c0 | | UDP-glycosyltransferase (UGT) | | 3.3354 | 4.1564 | 6.9015 |
| c100473.graph_c0 | | Cytochrome P450 | | 50.5999 | 20.0065 | 70.6451 |
| c101334.graph_c0 | | Cytochrome P450 | | 3.4469 | 6.8025 | 0.0399 |
| c101377.graph_c0 | | Cytochrome P450 | | 7.4999 | 0.8157 | 0.8044 |
| c102160.graph_c0 | | Cytochrome P450 | | 0.4911 | 10.5980 | 6.2228 |
| c102246.graph_c0 | | Cytochrome P450 | | 0.9471 | 2.8740 | 7.0353 |
| c102487.graph_c0 | | Cytochrome P450 | | 0.0076 | 4.8840 | 0.0000 |
| c102978.graph_c0 | | Cytochrome P450 | | 0.4062 | 24.1178 | 3.1225 |
| c103884.graph_c0 | | Cytochrome P450 | | 51.6188 | 6.8747 | 22.1951 |
| c104177.graph_c0 | | Cytochrome P450 | | 3.1252 | 0.5783 | 1.6448 |
| c104264.graph_c0 | | Cytochrome P450 | | 25.7514 | 4.2529 | 5.0011 |
| c104780.graph_c0 | | Cytochrome P450 | | 9.1517 | 20.1387 | 0.5173 |
| c106335.graph_c0 | | Cytochrome P450 | | 158.7014 | 168.3037 | 509.5306 |
| c106616.graph_c0 | | Cytochrome P450 | | 282.1309 | 94.4984 | 283.0051 |
| c106981.graph_c4 | | Cytochrome P450 | | 220.9548 | 206.4897 | 23.9197 |
| c106981.graph_c5 | | Cytochrome P450 | | 191.7299 | 145.2615 | 22.0718 |
| c107281.graph_c0 | | Cytochrome P450 | | 869.5904 | 117.2664 | 410.4409 |
| c107953.graph_c0 | | Cytochrome P450 | | 14.6026 | 0.3359 | 10.9822 |
| c108136.graph_c0 | | Cytochrome P450 | | 30.6594 | 10.3035 | 4.4496 |
| c109094.graph_c0 | | Cytochrome P450 | | 29.3965 | 16.1474 | 10.1620 |
| c109206.graph_c0 | | Cytochrome P450 | | 1.1803 | 42.9106 | 3.4500 |
| c111829.graph_c0 | | Cytochrome P450 | | 2.3783 | 22.6909 | 0.6124 |
| c112043.graph_c0 | | Cytochrome P450 | | 7.9219 | 0.8277 | 4.3629 |
| c113123.graph_c0 | | Cytochrome P450 | | 2.7468 | 1.5859 | 4.3975 |
| c114312.graph_c0 | | Cytochrome P450 | | 1.5335 | 5.5066 | 0.0484 |
| c25548.graph_c0 | | Cytochrome P450 | | 3.8437 | 0.9741 | 24.9484 |
| c25788.graph_c0 | | Cytochrome P450 | | 9.1748 | 7.8755 | 43.9449 |
| c26254.graph_c0 | | Cytochrome P450 | | 3.6450 | 3.6340 | 8.2733 |
| c27475.graph_c0 | | Cytochrome P450 | | 3.3847 | 32.0405 | 0.9444 |
| c27730.graph_c0 | | Cytochrome P450 | | 0.1507 | 44.7385 | 1.4348 |
| c28645.graph_c0 | | Cytochrome P450 | | 18.0465 | 35.1399 | 52.7506 |
| c34065.graph_c0 | | Cytochrome P450 | | 0.0690 | 6.6218 | 0.8936 |
| c36159.graph_c0 | | Cytochrome P450 | | 0.0121 | 9.1442 | 1.4712 |
| c41493.graph_c0 | | Cytochrome P450 | | 0.0748 | 2.2626 | 4.1111 |
| c44662.graph_c0 | | Cytochrome P450 | | 16.1818 | 10.0091 | 8.7498 |
| c49533.graph_c0 | | Cytochrome P450 | | 86.7591 | 47.8685 | 57.6434 |
| c51727.graph_c0 | | Cytochrome P450 | | 0.0859 | 103.1131 | 8.8097 |
| c52663.graph_c1 | | Cytochrome P450 | | 33.4042 | 22.6859 | 14.3339 |
| c59764.graph_c1 | | Cytochrome P450 | | 2.8371 | 0.0402 | 0.6080 |
| c65649.graph_c0 | | Cytochrome P450 | | 0.3145 | 13.6761 | 11.7633 |
| c67372.graph_c1 | | Cytochrome P450 | | 0.1506 | 93.7997 | 7.4303 |
| c67531.graph_c0 | | Cytochrome P450 | | 30.8542 | 17.0062 | 131.0859 |
| c70096.graph_c0 | | Cytochrome P450 | | 0.0735 | 14.0625 | 1.5983 |
| c70339.graph_c0 | | Cytochrome P450 | | 0.1345 | 12.2983 | 0.6226 |
| c70477.graph_c0 | | Cytochrome P450 | | 0.0181 | 8.7905 | 2.2305 |
| c70652.graph_c0 | | Cytochrome P450 | | 0.0144 | 8.9584 | 0.7113 |
| c71405.graph_c0 | | Cytochrome P450 | | 0.1909 | 1.3144 | 4.8317 |
| c71770.graph_c0 | | Cytochrome P450 | | 0.0000 | 15.4256 | 2.0041 |
| c80418.graph_c0 | | Cytochrome P450 | | 5.8582 | 6.7864 | 0.0111 |
| c82002.graph_c0 | | Cytochrome P450 | | 0.1667 | 17.3250 | 1.8486 |
| c82508.graph_c0 | | Cytochrome P450 | | 55.9575 | 41.0148 | 17.1647 |
| c86380.graph_c0 | | Cytochrome P450 | | 0.0133 | 0.2186 | 3.5860 |
| c89392.graph_c0 | | Cytochrome P450 | | 14.1168 | 2.1108 | 2.8754 |
| c91264.graph_c0 | | Cytochrome P450 | | 0.4697 | 9.3178 | 0.1847 |
| c93755.graph_c0 | | Cytochrome P450 | | 0.0000 | 4.3401 | 0.0740 |
| c94828.graph_c0 | | Cytochrome P450 | | 1.7505 | 22.7499 | 9.7079 |
| c95499.graph_c0 | | Cytochrome P450 | | 0.0000 | 14.5922 | 0.5959 |
| c96387.graph_c0 | | Cytochrome P450 | | 0.1495 | 33.1081 | 2.1715 |
| c96526.graph_c0 | | Cytochrome P450 | | 16.2594 | 14.2817 | 4.8971 |
| c96799.graph_c0 | | Cytochrome P450 | | 0.9428 | 0.5914 | 5.6356 |
| c96855.graph_c0 | | Cytochrome P450 | | 0.4926 | 12.0968 | 0.2712 |
| c96953.graph_c0 | | Cytochrome P450 | | 12.2109 | 23.5410 | 35.3309 |
| c98618.graph_c0 | | Cytochrome P450 | | 0.1202 | 0.4706 | 1.4436 |
| c99086.graph_c0 | | Cytochrome P450 | | 17.9977 | 12.4936 | 6.6142 |
| c99718.graph_c1 | | Cytochrome P450 | | 2.6705 | 0.9424 | 1.6894 |
| c105703.graph_c0 | | O-methyltransferase (OMT) | | 1.8690 | 52.7115 | 18.3097 |
| c111467.graph_c0 | | O-methyltransferase (OMT) | | 0.8740 | 0.5100 | 16.8662 |
| c44733.graph_c0 | | O-methyltransferase (OMT) | | 0.0512 | 87.7115 | 2.6223 |
| c54346.graph_c0 | | O-methyltransferase (OMT) | | 0.0868 | 10.7434 | 1.8374 |
| c80107.graph_c0 | | O-methyltransferase (OMT) | | 0.8044 | 4.3365 | 10.7340 |
| c83052.graph_c0 | | O-methyltransferase (OMT) | | 2.9941 | 1.3181 | 0.3093 |
| c91204.graph_c0 | | O-methyltransferase (OMT) | | 113.3525 | 245.3149 | 22.1243 |
| c92198.graph_c0 | | O-methyltransferase (OMT) | | 0.1906 | 292.8041 | 20.3630 |
| c95465.graph_c0 | | O-methyltransferase (OMT) | | 0.0593 | 5.5935 | 1.0207 |
| **Transporter-related genes** | | | | | | |
| c102118.graph_c0 | | ABC transporter | | 7.5996 | 0.5698 | 7.8524 |
| c82796.graph_c0 | | ABC transporter | | 0.1221 | 9.7820 | 4.3469 |
| c107761.graph_c0 | | ABC transporter | | 27.4818 | 16.5528 | 93.9048 |
| c90873.graph_c0 | | ABC transporter | | 0.2069 | 16.8966 | 2.1404 |
| c105233.graph_c0 | | ABC transporter | | 4.3855 | 45.3153 | 10.6862 |
| c106606.graph_c0 | | ABC transporter | | 0.0265 | 0.3660 | 1.5967 |
| c106742.graph_c0 | | ABC transporter | | 4.2940 | 60.1733 | 17.5815 |
| c106078.graph_c1 | | ABC transporter | | 0.2540 | 10.2366 | 2.6108 |
| c27484.graph_c0 | | ABC transporter | | 0.9674 | 5.9515 | 1.9869 |
| c101413.graph_c0 | | ABC transporter | | 0.8924 | 7.8936 | 0.3352 |
| c95670.graph_c0 | | ABC transporter | | 0.0635 | 1.4820 | 0.0000 |
| c88399.graph_c0 | | ABC transporter | | 97.5142 | 30.7436 | 21.7664 |
| c87778.graph_c0 | | ABC transporter | | 2.2567 | 10.7731 | 70.9575 |
| c82379.graph_c0 | | ABC transporter | | 34.9281 | 5.3734 | 9.7549 |
| c109060.graph_c0 | | ABC transporter | | 15.5025 | 3.4518 | 18.3320 |
| c28046.graph_c0 | | ABC transporter | | 3.7692 | 21.6748 | 119.5852 |
| c70143.graph_c1 | | ABC transporter | | 0.0000 | 4.8940 | 0.0000 |
| c110139.graph_c0 | | ABC transporter | | 2.3206 | 18.6510 | 6.1020 |
| c101560.graph_c0 | | ABC transporter | | 0.5727 | 3.0352 | 1.0666 |
| c70143.graph_c0 | | ABC transporter | | 0.0000 | 6.0660 | 0.0000 |
| c98739.graph_c1 | | ABC transporter | | 0.5385 | 5.1283 | 1.3022 |
| c95136.graph_c1 | | ABC transporter | | 12.5300 | 9.2034 | 1.8337 |
| c102185.graph_c0 | | ABC transporter | | 0.6080 | 3.2045 | 0.4668 |
| c99814.graph_c0 | | ABC transporter | | 0.5474 | 0.1306 | 0.0062 |
| c67084.graph_c0 | | ABC transporter | | 0.1066 | 6.8208 | 6.1879 |
| c88399.graph_c1 | | ABC transporter | | 139.9574 | 40.6897 | 33.0008 |
| c104857.graph_c0 | | ABC transporter | | 1.8258 | 9.6959 | 3.2027 |
| c60943.graph_c0 | | ABC transporter | | 0.0467 | 2.3197 | 0.9724 |
| c69480.graph_c0 | | ABC transporter | | 181.2387 | 49.1189 | 48.3905 |
| c95136.graph_c0 | | ABC transporter | | 12.0446 | 8.5431 | 2.1112 |
| c43205.graph_c0 | | ABC transporter | | 0.8534 | 12.4166 | 10.3873 |
| c105122.graph_c0 | | ABC transporter | | 0.3311 | 3.8122 | 3.4896 |
| c102649.graph_c2 | | MRP transporter | | 1.5205 | 11.0840 | 9.8196 |
| c99512.graph_c0 | | MRP transporter | | 9.4583 | 1.0409 | 4.7131 |
| c71132.graph_c0 | | MRP transporter | | 0.3566 | 3.1912 | 2.5175 |
| c95670.graph_c1 | | MRP transporter | | 0.0738 | 2.4559 | 0.0542 |
| c91088.graph_c1 | | MRP transporter | | 4.4959 | 13.6741 | 38.4321 |
| c106379.graph_c4 | | MRP transporter | | 6.6815 | 6.3304 | 26.2376 |
| c112376.graph_c0 | | MRP transporter | | 0.2797 | 8.1106 | 1.3573 |
| c46354.graph_c0 | | MRP transporter | | 44.8684 | 31.8420 | 184.6323 |
| c28154.graph_c0 | | H+-ATPase | | 2.5867 | 20.3119 | 6.6468 |
| c47895.graph_c0 | | H+-ATPase | | 1.6332 | 6.4644 | 7.7855 |
| c38083.graph_c0 | | H+-ATPase | | 2.1661 | 0.5262 | 1.7971 |
| c17925.graph_c0 | | Multidrug and toxic compound extrusion protein (MATE) | | 0.4803 | 2.5251 | 1.6708 |
| c86637.graph_c0 | | Multidrug and toxic compound extrusion protein (MATE) | | 0.1563 | 0.6105 | 1.4804 |
| c105476.graph_c0 | | Multidrug and toxic compound extrusion protein (MATE) | | 0.4051 | 5.1555 | 0.0762 |
| c105426.graph_c0 | | Multidrug and toxic compound extrusion protein (MATE) | | 1.1240 | 2.4478 | 0.3363 |
| c101867.graph_c0 | | Multidrug and toxic compound extrusion protein (MATE) | | 5.8712 | 57.1449 | 0.4014 |
| c104949.graph_c7 | | Multidrug and toxic compound extrusion protein (MATE) | | 19.5135 | 10.3181 | 68.9066 |
| c98204.graph_c0 | | Multidrug and toxic compound extrusion protein (MATE) | | 16.6204 | 4.3868 | 5.0645 |
| c44952.graph_c0 | | Multidrug and toxic compound extrusion protein (MATE) | | 0.0590 | 0.7996 | 1.5115 |
| c100621.graph_c0 | | Multidrug and toxic compound extrusion protein (MATE) | | 24.2795 | 5.8179 | 31.7230 |
| c94621.graph_c0 | | Glutathione S-transferase (GST) | | 25.2329 | 7.3037 | 6.7297 |
| c82166.graph_c1 | | Glutathione S-transferase (GST) | | 0.4458 | 0.8729 | 4.6675 |
| c93866.graph_c0 | | Glutathione S-transferase (GST) | | 2.1683 | 78.1196 | 38.5561 |
| c110117.graph_c0 | | Glutathione S-transferase (GST) | | 1.8672 | 2.3304 | 11.3767 |
| c95235.graph_c0 | | Glutathione S-transferase (GST) | | 35.4902 | 7.3538 | 10.8577 |
| c69253.graph_c0 | | Glutathione S-transferase (GST) | | 1.0688 | 3.6358 | 8.1450 |
| c107525.graph_c0 | | Glutathione S-transferase (GST) | | 0.7877 | 13.9931 | 608.4138 |
| c91710.graph_c1 | | Glutathione S-transferase (GST) | | 19.1840 | 4.8211 | 20.2426 |
| c73626.graph_c1 | | Glutathione S-transferase (GST) | | 18.2602 | 3.1027 | 1.7566 |
| c24517.graph_c0 | | Glutathione S-transferase (GST) | | 21.6294 | 14.3953 | 71.2579 |
| c24974.graph_c0 | | Glutathione S-transferase (GST) | | 1.1229 | 1.5558 | 10.8249 |
| **Transcription factors** | | | |  |  |  |
| c102218.graph_c1 | | MYB transcription factor | | 0.6158 | 4.2151 | 3.3125 |
| c102770.graph_c0 | | MYB transcription factor | | 0.5020 | 3.4689 | 1.7070 |
| c110122.graph_c0 | | MYB transcription factor | | 1.6249 | 10.9990 | 2.7552 |
| c110732.graph_c0 | | MYB transcription factor | | 0.5067 | 0.9626 | 10.8854 |
| c112979.graph_c0 | | MYB transcription factor | | 0.9707 | 2.6301 | 0.5733 |
| c114362.graph_c0 | | MYB transcription factor | | 0.5509 | 3.1153 | 0.3975 |
| c114666.graph_c0 | | MYB transcription factor | | 0.7122 | 0.4256 | 2.6647 |
| c115433.graph_c0 | | MYB transcription factor | | 0.0114 | 2.7759 | 0.1643 |
| c117355.graph_c0 | | MYB transcription factor | | 1.7180 | 0.2384 | 0.5319 |
| c22815.graph_c0 | | MYB transcription factor | | 0.3348 | 33.2498 | 6.3371 |
| c24525.graph_c0 | | MYB transcription factor | | 5.5548 | 81.6235 | 14.0302 |
| c27438.graph_c0 | | MYB transcription factor | | 1.7798 | 12.1382 | 3.3872 |
| c29272.graph_c0 | | MYB transcription factor | | 0.7788 | 0.2508 | 11.4808 |
| c46854.graph_c0 | | MYB transcription factor | | 1.3069 | 2.9233 | 30.0691 |
| c47321.graph_c0 | | MYB transcription factor | | 5.0661 | 1.2936 | 0.0666 |
| c51112.graph_c0 | | MYB transcription factor | | 0.0483 | 0.1547 | 49.0865 |
| c53045.graph_c0 | | MYB transcription factor | | 0.0000 | 1.2672 | 0.1347 |
| c67482.graph_c0 | | MYB transcription factor | | 1.0534 | 6.1372 | 1.4652 |
| c67676.graph_c0 | | MYB transcription factor | | 13.0599 | 61.8800 | 8.8983 |
| c68332.graph_c0 | | MYB transcription factor | | 0.2775 | 2.1476 | 15.5091 |
| c69715.graph_c0 | | MYB transcription factor | | 18.0491 | 2.5432 | 4.0114 |
| c70746.graph_c0 | | MYB transcription factor | | 29.9332 | 37.0049 | 6.1970 |
| c70946.graph_c0 | | MYB transcription factor | | 0.7272 | 3.8652 | 0.9198 |
| c71176.graph_c0 | | MYB transcription factor | | 0.4440 | 0.2091 | 2.4057 |
| c71945.graph_c0 | | MYB transcription factor | | 3.1186 | 86.7544 | 5.9971 |
| c86195.graph_c0 | | MYB transcription factor | | 279.0223 | 50.9678 | 48.4296 |
| c89336.graph_c0 | | MYB transcription factor | | 0.0130 | 0.7971 | 0.1477 |
| c89370.graph_c0 | | MYB transcription factor | | 0.1232 | 0.9694 | 4.2135 |
| c92327.graph_c1 | | MYB transcription factor | | 0.4777 | 55.4083 | 11.6093 |
| c92730.graph_c0 | | MYB transcription factor | | 0.0166 | 6.1103 | 0.0311 |
| c93020.graph_c0 | | MYB transcription factor | | 0.0139 | 2.0923 | 0.2919 |
| c93061.graph_c0 | | MYB transcription factor | | 0.0000 | 4.1622 | 0.4814 |
| c96216.graph_c0 | | MYB transcription factor | | 0.9654 | 7.0850 | 1.8041 |
| c97255.graph_c0 | | MYB transcription factor | | 0.0250 | 2.3600 | 0.0745 |
| c100935.graph_c0 | | bHLH transcription factor | | 0.6422 | 0.7405 | 18.0618 |
| c108786.graph_c0 | | bHLH transcription factor | | 14.0445 | 51.7144 | 8.7587 |
| c24481.graph_c0 | | bHLH transcription factor | | 14.9014 | 77.2915 | 5.2411 |
| c25255.graph_c0 | | bHLH transcription factor | | 19.9494 | 201.9986 | 23.9804 |
| c26955.graph_c0 | | bHLH transcription factor | | 2.1658 | 17.4736 | 28.1709 |
| c36739.graph_c0 | | bHLH transcription factor | | 7.7934 | 0.0515 | 0.0000 |
| c42659.graph_c0 | | bHLH transcription factor | | 0.0236 | 5.6443 | 0.1762 |
| c50008.graph_c0 | | bHLH transcription factor | | 1.4869 | 38.2824 | 1.5513 |
| c54086.graph_c0 | | bHLH transcription factor | | 0.0146 | 6.4450 | 0.8605 |
| c71855.graph_c1 | | bHLH transcription factor | | 5.6696 | 74.9375 | 1.4887 |
| c86148.graph_c0 | | bHLH transcription factor | | 34.0235 | 352.7041 | 8.4359 |
| c89199.graph_c0 | | bHLH transcription factor | | 1.6012 | 0.3596 | 0.9877 |
| c90870.graph_c0 | | bHLH transcription factor | | 2.3938 | 6.5655 | 0.9502 |
| c91715.graph_c0 | | bHLH transcription factor | | 0.5819 | 1.6773 | 300.5862 |
| c92233.graph_c0 | | bHLH transcription factor | | 9.8657 | 64.6765 | 35.3525 |
| c92960.graph_c0 | | bHLH transcription factor | | 2.2866 | 3.2178 | 32.1822 |
| c93897.graph_c0 | | bHLH transcription factor | | 11.0654 | 7.8648 | 1.3920 |
| c96140.graph_c0 | | bHLH transcription factor | | 0.5730 | 17.2809 | 0.9820 |
| c96997.graph_c0 | | bHLH transcription factor | | 6.1163 | 10.5799 | 67.3283 |
| c98850.graph_c0 | | bHLH transcription factor | | 13.4272 | 126.5833 | 16.2811 |
| c99851.graph_c0 | | bHLH transcription factor | | 0.6808 | 5.0716 | 0.4400 |
| c105703.graph_c0 | | WD40 transcription factor | | 1.8690 | 17.5705 | 18.3097 |
| c111467.graph_c0 | | WD40 transcription factor | | 0.8740 | 0.1700 | 16.8662 |
| c44733.graph_c0 | | WD40 transcription factor | | 0.0512 | 29.2372 | 2.6223 |
| c54346.graph_c0 | | WD40 transcription factor | | 0.0868 | 3.5811 | 1.8374 |
| c80107.graph_c0 | | WD40 transcription factor | | 0.8044 | 1.4455 | 10.7340 |
| c83052.graph_c0 | | WD40 transcription factor | | 2.9941 | 0.4394 | 0.3093 |
| c91204.graph_c0 | | WD40 transcription factor | | 113.3525 | 81.7716 | 22.1243 |
| c92198.graph_c0 | | WD40 transcription factor | | 0.1906 | 97.6014 | 20.3630 |
| c95465.graph_c0 | | WD40 transcription factor | | 0.0593 | 1.8645 | 1.0207 |
