# Supplementary material for: Comparative transcriptome among Euscaphis konishii Hayata tissues and analysis of genes involved in flavonoid biosynthesis and accumulation
Source: BMC Genomics. 2019 Jan 9;20:24. doi: 10.1186/s12864-018-5354-x (PMC6327468; doi:10.1186/s12864-018-5354-x)
Supplement: Supplementary file 5 — Phylogenetic tree analysis of Candidate genes involved in Flavonoid biosynthesis (DOCX 15761 kb) [file 12864_2018_5354_MOESM5_ESM.docx]

**
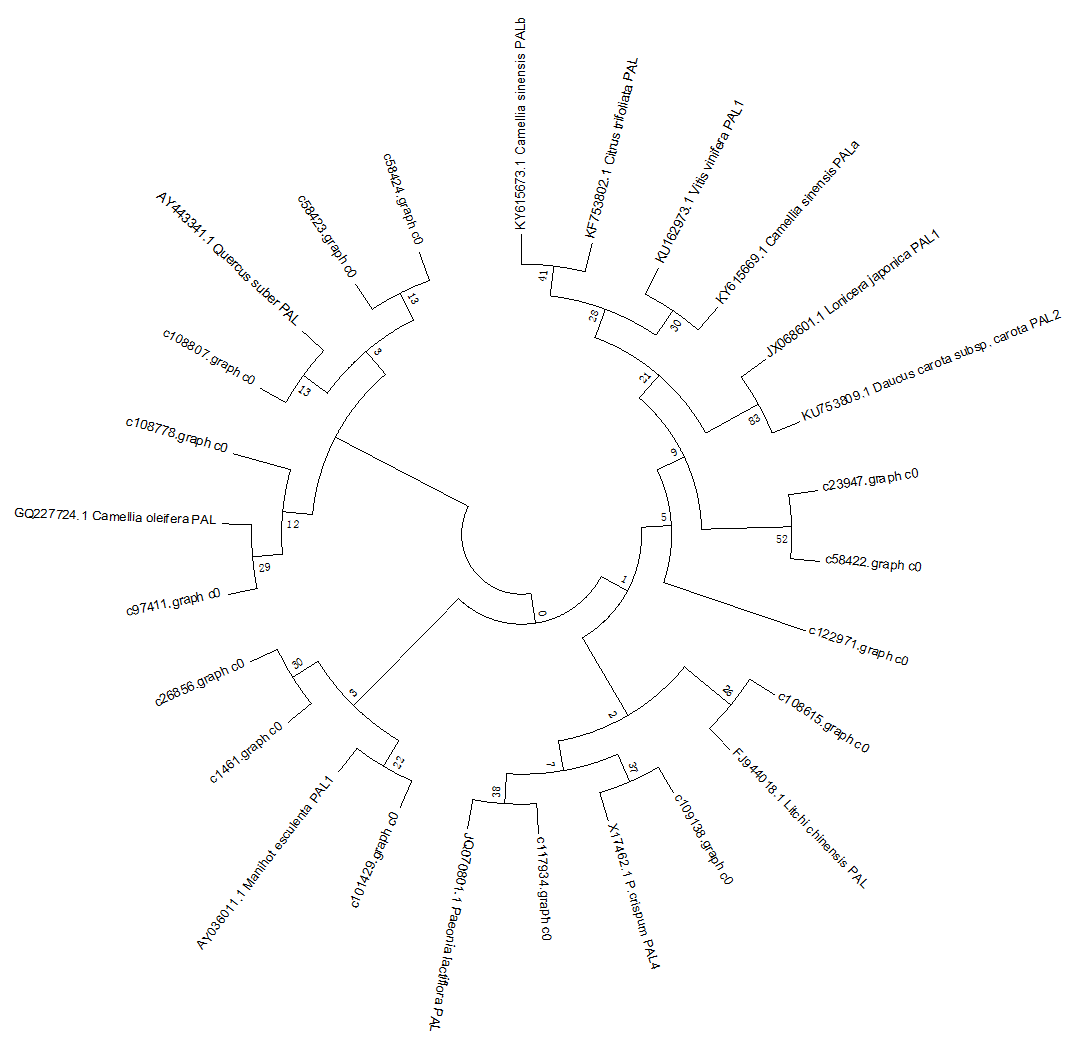
**

**Figure S2.** Phylogenetic tree of *E. konishii* Phenylalanine ammonia lyse (*PAL*). **(**The evolutionary history was inferred using the Neighbor-Joining method. The bootstrap consensus tree inferred from 1000 replicates is taken to represent the evolutionary history of the taxa analyzed. Branches corresponding to partitions reproduced in less than 50% bootstrap replicates are collapsed. The percentage of replicate trees in which the associated taxa clustered together in the bootstrap test (1000 replicates) are shown next to the branches. The evolutionary distances were computed using the Maximum Composite Likelihood method and are in the units of the number of base substitutions per site. Evolutionary analyses were conducted in MEGA7. And the same was below.**)**

**
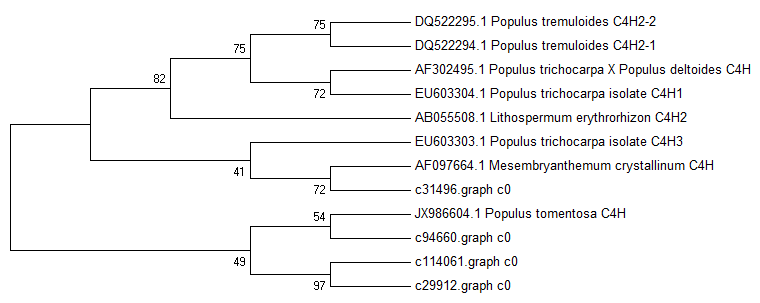
**

**Figure S3.** Phylogenetic tree of *E. konishii* Cinnamate-4-hydroxylase (*C4H*)


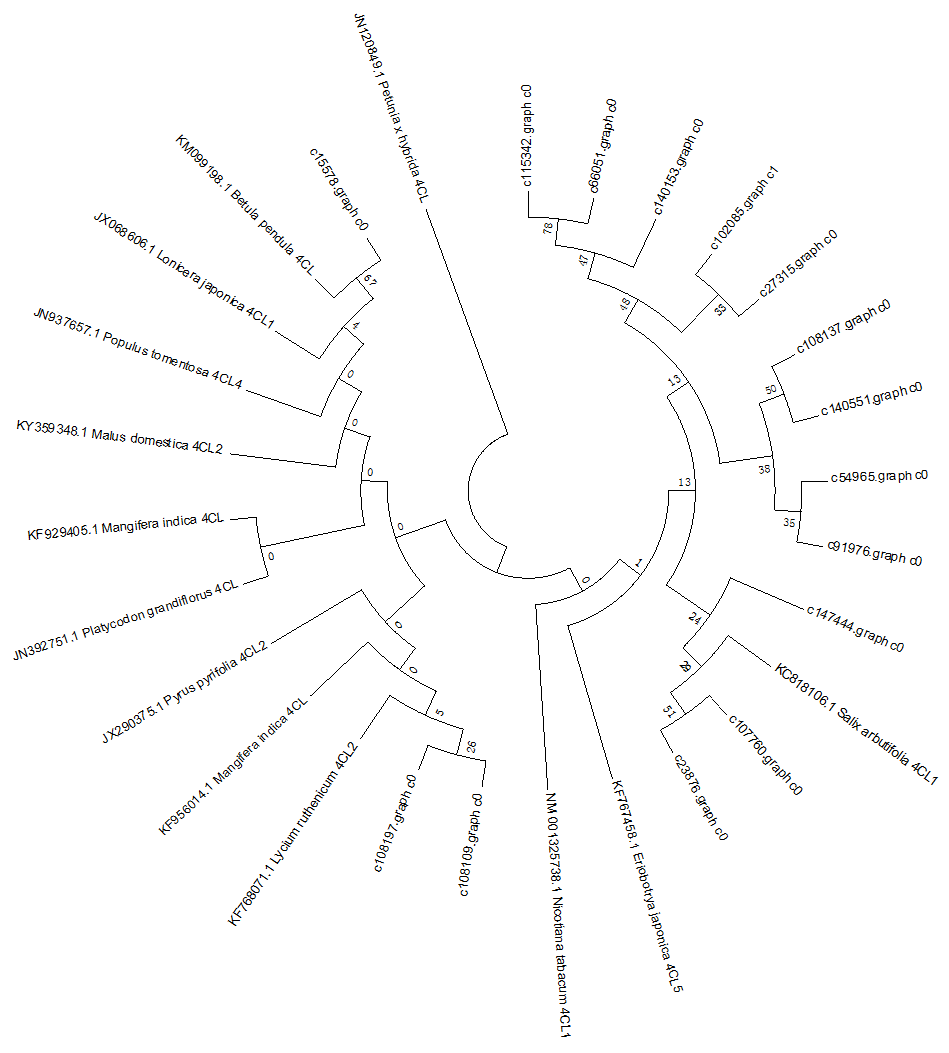


**Figure S4.** Phylogenetic tree of *E. konishii* 4-coumarate CoA ligase (*4CL*)


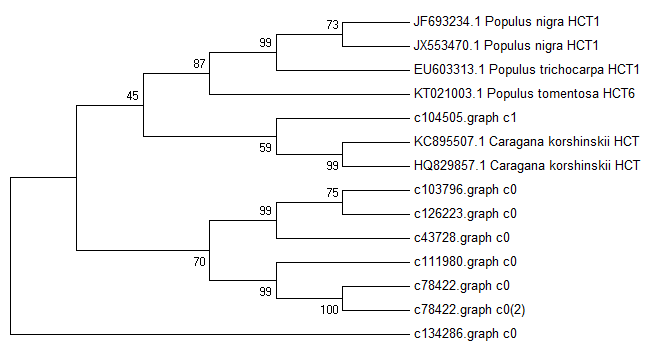


**Figure S5.** Phylogenetic tree of *E. konishii* Shikimate O-hydroxycinnamoyltransferase (*HCT*)

**
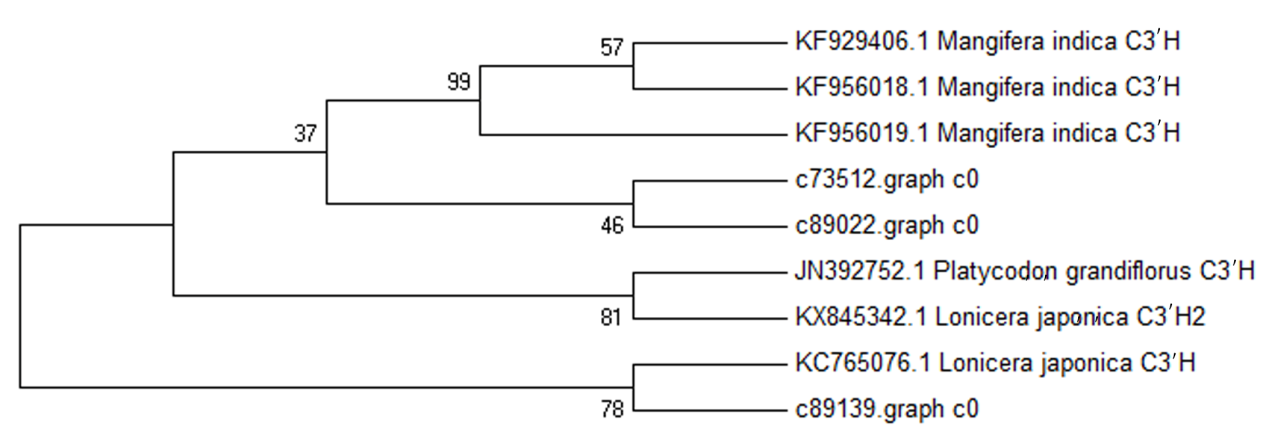
**

**Figure S6.** Phylogenetic tree of *E. konishii* Coumaroylquinate (coumaroylshikimate) 3'-

monooxygenase (*C3'H*)

**
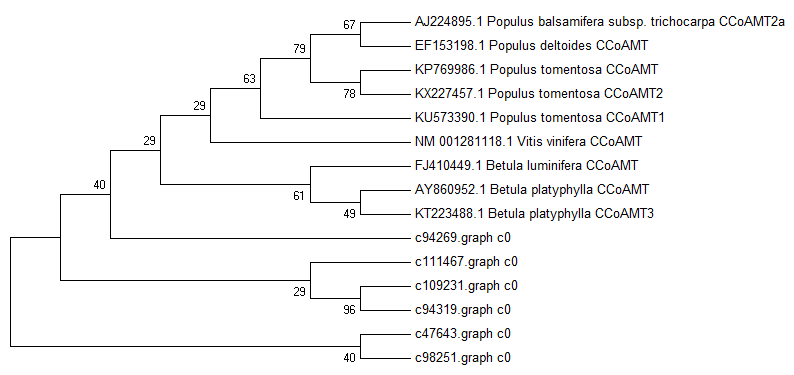
**

**Figure S7.** Phylogenetic tree of *E. konishii* Caffeoyl-CoA O-methyltransferase (*CCoAMT*)

**
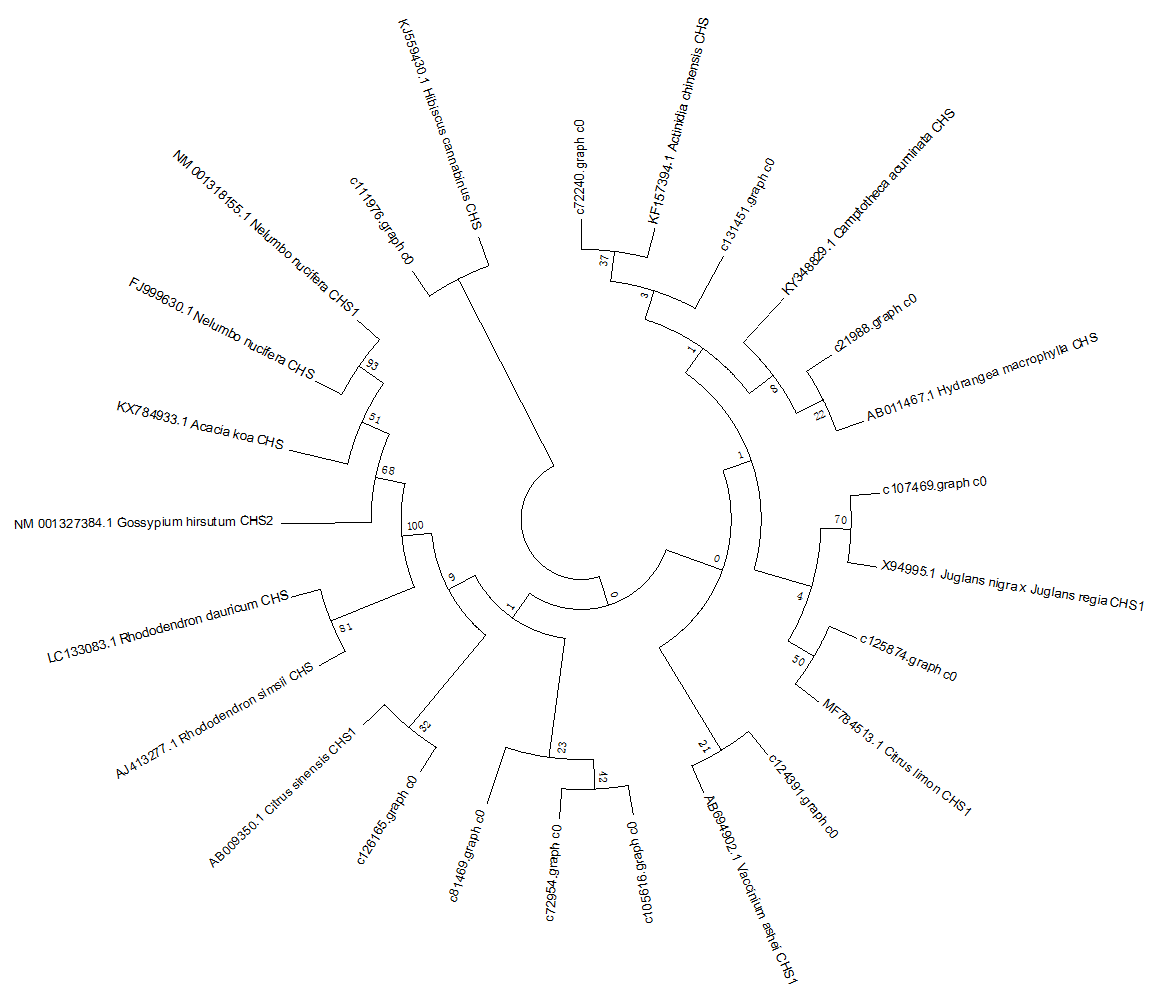
**

**Figure S8.** Phylogenetic tree of *E. konishii* Chalcone synthase (*CHS*)

**
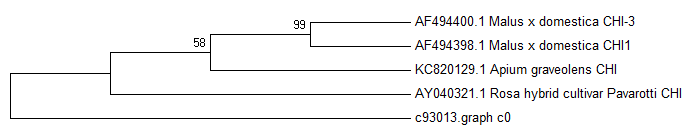
**

**Figure S9.** Phylogenetic tree of *E. konishii* Chalcone isomerase (*CHI*)


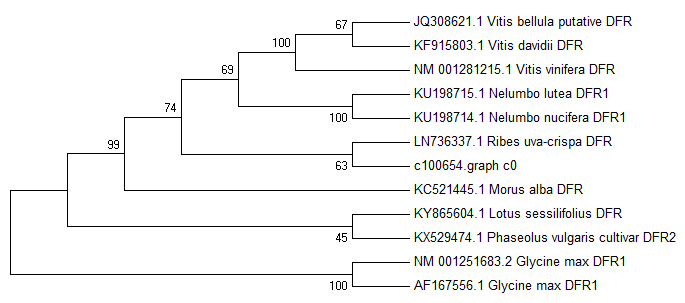


**Figure S10.** Phylogenetic tree of *E. konishii* hydroflavonol 4-reductase (*DFR*)

**
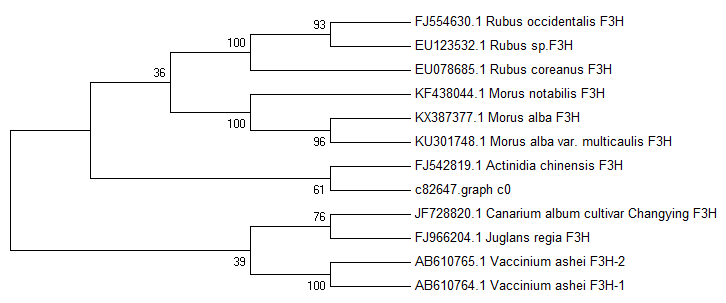
**

**Figure S11. Phylogenetic tree of *E. konishii* Flavanone-3-hydroxylase (*F3H*)**


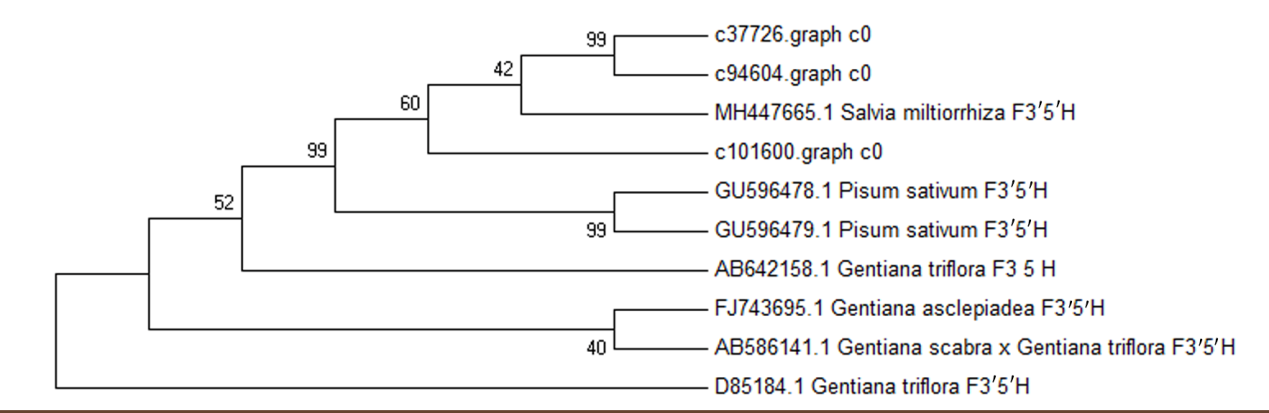


**Figure S12. Phylogenetic tree of *E. konishii* Flavonoid-3'5'-hydroxylase (*F3'5'H*)**

**
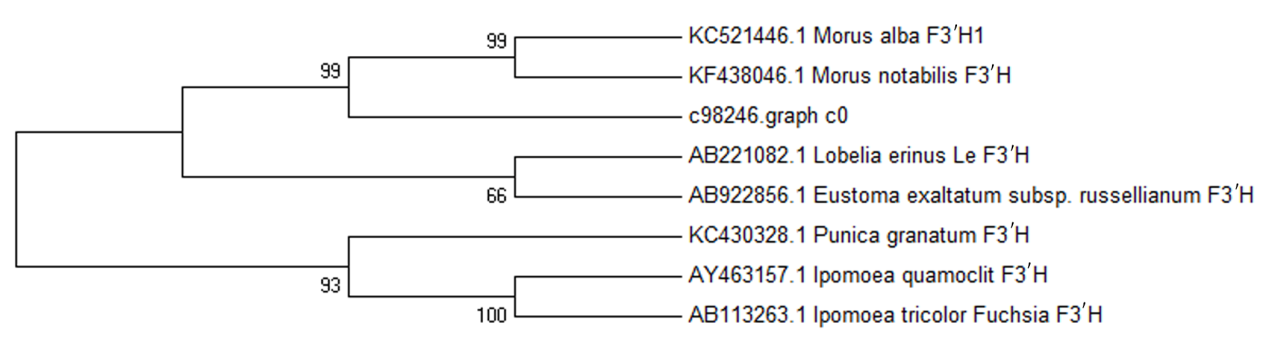
**

**Figure S13. Phylogenetic tree of *E. konishii* Flavonoid-3'-hydroxylase (*F3'H*)**


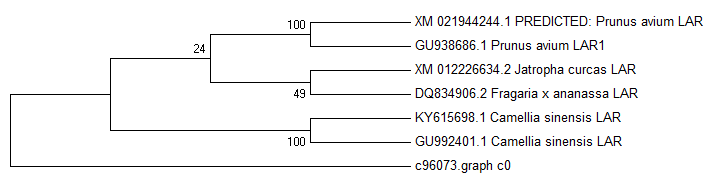


**Figure S14. Phylogenetic tree of *E. konishii* Leucoanthocyanidin reductase (*LAR*)**

**
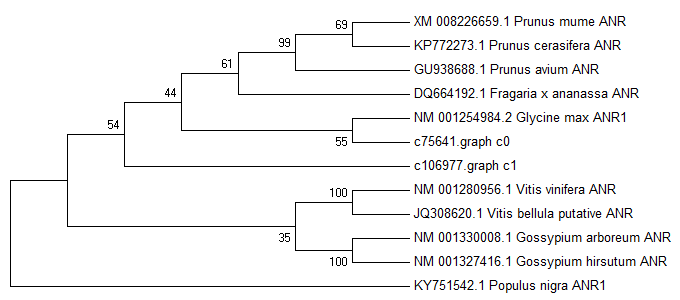
**

**Figure S15. Phylogenetic tree of *E. konishii* Anthocyanidin reductase (*ANR*)**


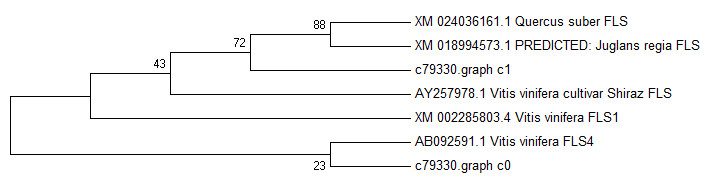


**Figure S16. Phylogenetic tree of *E. konishii* Flaconol synthase (*FLS*)**


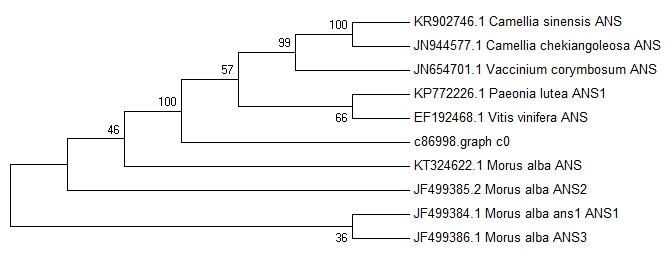


**Figure S16. Phylogenetic tree of *E. konishii* Anthocyanidin synthase (*ANS*)**
