# Supplementary material for: Comparative transcriptome among Euscaphis konishii Hayata tissues and analysis of genes involved in flavonoid biosynthesis and accumulation
Source: BMC Genomics. 2019 Jan 9;20:24. doi: 10.1186/s12864-018-5354-x (PMC6327468; doi:10.1186/s12864-018-5354-x)
Supplement: Supplementary file 6 — Primers used in validation experiment of gene expression by qRT-PCR (DOCX 13 kb) [file 12864_2018_5354_MOESM6_ESM.docx]

**Table S4. Primers used in validation experiment of gene expression by qRT-PCR**

| Gene id | Forward primer | Reverse primer |
| --- | --- | --- |
| c103858.graph_c0 | AGTCCCTTTGTCTGGAAAGATG | CTCATCCGTCACCTTCTCATTT |
| c111980.graph_c0 | GACTCGAGGAGAGAGTGTAGAT | CTGGAATTCCTCGTGGTCATAG |
| c89139.graph_c0 | GTGAAGGTGAGGAAGGTTTGTA | GGTTACTGCTATGGTTGAGTCC |
| c111467.graph_c0 | CGCCGACAAAGAGAACTACAT | TTATGGTTCGGCTTAGTTGCA |
| c47643.graph_c0 | GGCTCTTCCGGTTCTTGATAA | TATCACGAGAGGCTGATGAAAC |
| c105616.graph_c0 | GCCAAACGAGAATGGACAATATG | TCTTGTTCCTTCGGGTGATG |
| c107469.graph_c0 | GGAACTCGCTGTTCTGGATAG | CCTTGTGGCCCTTAACTTCT |
| c111976.graph_c0 | GATGGGTTGGCTCCATTCTT | GAAAGAAGGAGCCTTGTAGGTT |
| c93013.graph_c0 | CCCAGTAAAGGAAGACCAAGAA | TAGTTTCTCTTGTCCAGCAGC |
| c100654.graph_c0 | CCTGGATAGTTGGGTGAACAA | AGATAGCCCAGTTAATTCGGAC |
| c82647.graph_c0 | ATTCCCTACCAACCAACCTTC | GATACAGGGTTCGGGACATTT |
| c101600.graph_c0 | AGTTCGAGCACAAGAGCTAAA | GTATCGGAGATGTTGACGTACG |
| c96073.graph_c0 | CCATCGGAGTTTGGACATGATA | GTACACCTGACTCCTCAATCAC |
| c79330.graph_c0 | TCTCGGACATGGTGGGTAATA | ATGTTCTTCAAGTCCTGCCC |
| c86998.graph_c0 | TGGTTTGGGACTGGAAGAAG | AACTACTATCCCAAATGCCCTC |
